# From Lab to Life: Self‐Powered Sweat Sensors and Their Future in Personal Health Monitoring

**DOI:** 10.1002/advs.202409178

**Published:** 2024-10-28

**Authors:** Nan Gao, Guodong Xu, Gang Chang, Yuxiang Wu

**Affiliations:** ^1^ Institute of Intelligent Sport and Proactive Health Department of Health and Physical Education Jianghan University Wuhan 430056 China; ^2^ Ministry of Education Key Laboratory for the Green Preparation and Application of Functional Materials Hubei Key Laboratory of Polymer Materials School of Materials Science and Engineering Hubei University No.368 Youyi Avenue, Wuchang Wuhan 430062 China

**Keywords:** energy harvester, health monitoring, prospect, self‐powered, sweat sensor

## Abstract

The rapid development of wearable sweat sensors has demonstrated their potential for continuous, non‐invasive disease diagnosis and health monitoring. Emerging energy harvesters capable of converting various environmental energy sources—biomechanical, thermal, biochemical, and solar—into electrical energy are revolutionizing power solutions for wearable devices. Based on self‐powered technology, the integration of the energy harvesters with wearable sweat sensors can drive the device for biosensing, signal processing, and data transmission. As a result, self‐powered sweat sensors are able to operate continuously without external power or charging, greatly facilitating the development of wearable electronics and personalized healthcare. This review focuses on the recent advances in self‐powered sweat sensors for personalized healthcare, covering sweat sensors, energy harvesters, energy management, and applications. The review begins with the foundations of wearable sweat sensors, providing an overview of their detection methods, materials, and wearable devices. Then, the working mechanism, structure, and a characteristic of different types of energy harvesters are discussed. The features and challenges of different energy harvesters in energy supply and energy management of sweat sensors are emphasized. The review concludes with a look at the future prospects of self‐powered sweat sensors, outlining the trajectory of the field and its potential to flourish.

## Graphical abstract

This review focuses on recent advances in self‐powered sweat sensors in personal health monitoring, including sweat sensors, energy harvesters, energy management, and their applications. It analyzes the difficulties and challenges in the research field of self‐powered sweat sensors and looks into the future direction of self‐powered sweat sensors from lab to life.

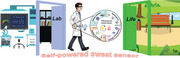

## 1 Introduction

Currently, wearable electronic devices are mainly used to measure physiological signs such as heart rate, body temperature, and blood pressure, which lack direct information for monitoring the dynamic biochemical and metabolic processes of the human body and are unable to provide a deeper level of biomolecular status.^[^ ^1^, ^2^, ^3^ ^]^ Sweat contains a variety of health‐related biomarkers, ranging from electrolytes and metabolites to proteins, cytokines, antigens, and exogenous drugs, reflecting an individual's comprehensive biomolecular state and level of health.^[^ ^4^, ^5^, ^6^ ^]^ For example, Na^+^, K^+,^ and Ca^2+^ are some of the main components of sweat, and their content reflects the loss of electrolytes in human activities^[^ ^7^, ^8^, ^9^ ^]^; The pH in sweat demonstrates the state of hydration in the body.^[^ ^10^ ^]^ Lactate, an essential indicator of physiological stress, reflects the body's transition from aerobic to anaerobic metabolism.^[^ ^11^, ^12^ ^]^ The concentration of glucose in sweat reveals the metabolism of blood sugar in the body.^[^ ^13^, ^14^ ^]^ The physiological information transmitted in human sweat is closely related to blood levels, and this non‐invasive detection method provides a unique advantage for the development of wearable electronics.^[^ ^15^, ^16^ ^]^ In addition, advances in detection technology, materials science, and flexible electronics are accelerating the boom in wearable sweat sensors.

Continuous sweat monitoring requires not only accurate and real‐time sensing devices but also a constant and efficient energy supply. Therefore, it is essential to develop renewable and sustainable power supplies.^[^ ^17^, ^18^, ^19^ ^]^ Recent advances in materials science and sensing technologies have given rise modern wearable electronic devices that harvest energy directly from the human body and the surrounding environment. Energy harvesters convert biomechanical energy, thermal energy, biochemical energy, and solar energy into electricity. Based on the working mechanism of the energy harvester, self‐powered technologies for sweat biosensors could be categorized into triboelectric nanogenerators,^[^ ^17^, ^20^, ^21^ ^]^ piezoelectric nanogenerators,^[^ ^22^, ^23^ ^]^ thermoelectric generators,^[^ ^24^, ^25^ ^]^ biofuel cells,^[^ ^26^, ^27^ ^]^ and photovoltaic cells.^[^ ^28^, ^29^ ^]^ Self‐powered sweat sensors, as an emerging wearable device, convert environmental energy into electricity through energy harvesting technology, enabling continuous monitoring of human health without external power sources. As a rusult, the self‐powered sweat sensors reduce the dependence on traditional batteries and are more suitable for wearable devices to achieve long‐term, continuous physiological monitoring. However, in practical applications, the collected energy of the self‐powered systems is not as efficient as that of the traditional battery power systems and is susceptible to environmental factors. To improve the energy efficiency and stability of self‐power supply systems, energy management techniques are particularly important. The efficient operation of self‐powered systems is achieved by optimizing energy harvesting, signal processing, wireless communication, and low‐power design.^[^ ^28^, ^30^, ^31^ ^]^ In addition, with the advancement of energy harvesting technology and material science, the limitations faced by self‐powered sweat sensors are expected to be overcome and would play a greater role in health monitoring and disease prevention.

**Figure 1:**
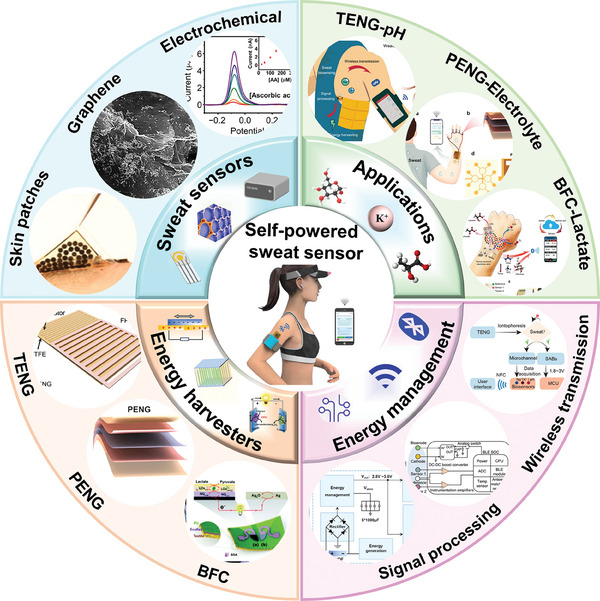
An overview of the self‐powered sweat sensors. (“Self‐powered sweat sensor”, Reproduced with permission.^[^ ^32^ ^]^ Copyright 2022, Wiley‐VCH GmbH; “Electrochemical”, Reproduced with permission.^[^ ^33^ ^]^ Copyright 2019, The American Association for the Advancement of Science. “Graphene”, Reproduced with permission.^[^ ^34^ ^]^ Copyright 2023, American Chemical Society. “Skin patches” Reproduced with permission.^[^ ^35^ ^]^ Copyright 2020, The American Association for the Advancement of Science; “TENG”, Reproduced with permission.^[^ ^36^ ^]^ Copyright 2020, The American Association for the Advancement of Science. “PENG”, Reproduced with permission.^[^ ^23^ ^]^ Copyright 2022, Elsevier. “BFC”, Reproduced with permission.^[^ ^37^ ^]^ Copyright 2016, The Royal Society of Chemistry; “Signal processing”, Reproduced with permission.^[^ ^23^ ^]^ Copyright 2022, Elsevier. Reproduced with permission.^[^ ^35^ ^]^ Copyright 2020, The American Association for the Advancement of Science. “Wireless transmission”, Reproduced with permission^[^ ^38^ ^]^ Copyright 2023, Wiley‐VCH GmbH; “BFC‐Lactate”, Reproduced with permission.^[^ ^35^ ^]^ Copyright 2020, The American Association for the Advancement of Science; “PENG‐Electrolyte”, Reproduced with permission.^[^ ^23^ ^]^ Copyright 2022, Elsevier. “TENG‐pH”, Reproduced with permission.^[^ ^36^ ^]^ Copyright 2020, The American Association for the Advancement of Science).

In this review, the recent research advances of self‐powered sweat‐sensing systems in health monitoring and human movement are systematically introduced and highlighted (**Figure** **1**). At the beginning of the review, the basic components of wearable sweat sensors are briefly discussed, including detection methods, materials, and wearable devices. Next, according to the wearable energy source, the energy harvesting devices are categorized into triboelectric nanogenerators, piezoelectric nanogenerators, thermoelectric generators, biofuel cells, and photovoltaic cells. The working mechanism, structural composition, and advantages and disadvantages of different types of self‐powered sensors are highlighted and analyzed. Then, the features and differences of different energy harvesters in wearable sweat‐sensing power supply and energy management units are emphasized. The signal processing and wireless transmission functions of the self‐powered sensor are also discussed. On this basis, applications of self‐powered sweat biosensors in personalized medicine and motion monitoring are summarized. Finally, an outlook on the future development of self‐powered wearable sensors from laboratory to life is prospected.

## 2 Wearable Sweat Sensors

Wearable sweat sensors allow non‐invasive and continuous monitoring of human health at the level of molecular information, which has received widespread attention in the field of personalized medicine. This section focuses on the analysis methods, nanomaterials, and wearable devices of sweat sensors. Section 2.1 describes the sensing mode and working mechanism of electrochemical and optical technology for wearable sweat sensors. Section 2.2 discusses the role of 0‐, 1‐ and 2D nanomaterials in sweat sensing. Section 2.3 analyzes the wearable devices of sweat sensor devices, including tattoos, skin patches, and textiles.

### 2.1 Sensing Technology of Sweat Sensors

#### 2.1.1 Electrochemical Mode

Electrochemical sensors are known for their high selectivity, excellent sensitivity, low response time, and easy adaptation to wearable devices.^[^ ^39^, ^40^ ^]^ Sweat biosensors are based on electrochemical mechanisms capable of providing molecular‐level information to indicate the health status of the body, and the commonly used methods are amperometry, potentiometric, voltammetry, and impedance methods. **Table** **1** summarizes the latest advances in sensing modes and performance of sweat sensors based on electrochemical principles.

**Table 1:** Wearable sweat sensors based on different electrochemical analysis modes.

Technique	Analyte	Materials	Detection Range	Sensitivity	LOD	Ref.
Amperometry	Glucose	Pt/MXene	0–1 mM	3.43 µA mM^−1^ cm^−2^	29.15 µM	[41]
	Glucose	MPA	0.05–1.4 mM	4.7 ± 0.8 µA mM^−1^	26 ± 5 µM	[42]
	Glucose	GOD‐GA‐Ni/Cu‐MOFs	0.001–20 mM	26.05 µA cm^−2^ mM^−1^	0.51 µM	[43]
	Glucose	NCGP	0.01–0.66 mM	425.9 µA mM^−1^·cm^−2^	3.28 µM	[44]
	Glucose	SilkNCT	25–300 µM	6.3 nA µM^−1^	5 µM	[33]
	Lactate		5–35 mM	174.0 nA mM^−1^	0.5 mM	
	Glucose	CNTs/Ti_3_C_2_T_x_/PB/CFMs	0.01–3 mM	35.3 µA mM^−1^ cm^−2^	0.33 µM	[45]
	Lactate		0–22 mM	11.4 µA mM^−1^ cm^−2^	0.67 µM	
	UA	BGQDs/CNTs	0–50 mM	8.92 ± 0.22 µA µM^−1^ cm^−2^	0.99 µM	[46]
	Glucose	Electrochemical fiber	50–300 µM	6 nA mM^−1^	4.55 µM	[10]
	Lactate		20–60 mM	69 nA mM^−1^	11.49 µM	
Potentiometric	Na^+^	SilkNCT	5–100 mM	51.8 mV decade^−1^	1 mM	[33]
	K^+^		1.25–40 mM	31.8 mV decade^−1^	0.5 mM	
	Lactate	ZnO NWs	0–25 mM	0.94 mV mM^−1^	3.61 mM	[47]
	Na^+^		0.1–100 mM	42.9 mV decade^−1^	0.16 mM	
	Na^+^	Solid contact‐ISE	0.01–10 mM	56.5 mV decade^−1^	1.67 µM	[48]
	K^+^		0.1–100 mM	65.7 mV decade^−1^	7.96 µM	
	Na^+^	Electrochemical fiber	10‐160 mM	67 mV decade^−1^	0.549 mM	[10]
	K^+^		2—32 mM	29.4 mV decade^−1^	0.211 mM	
	Ca^2+^		4–8 mM	39 mV decade^−1^	0.134 mM	
	pH		4–7	28 mV decade^−1^	**/**	
Voltammetry	AA	Alginate/CuO	10–150 µM	0.103 V log(µM)^−1^	1.97 µM	[49]
	Cl^−^	Silver ink on filter paper	10‐200 mM	1.98 µA mM^−1^	1 mM	[50]
	AA	SilkNCT	20–300 µM	22.7 nA µM^−1^	1 µM	[33]
	UA		2.5—115 µM	196.6 nA µM^−1^	0.1 µM	
	UA	LEG‐CS	0–200	3.50 µA µM^−1^ cm^−2^	0.74 µM	[51]
	Tyrosine		0–1000	0.61 µA µM^−1^ cm^−2^	3.6 µM	
	Phe	E‐MIP	10–300 µM	1.4 nA µM^−1^	4.7 µM	[52]
			300–1000 µM	0.27 nA µM^−1^		
	AA	Porous graphene	60–1000 µM	0.16 µA µM^−1 ^cm^−2^	30 µM	[53]
	UA		3–200 µM	6.87 µA µM^−1 ^cm^−2^	0.78 µM	
	DA		3–100 µM	6.49 µA µM^−1 ^cm^−2^	0.78 µM	
	Tyrosine		30–1000 µM	0.94 µA µM^−1 ^cm^−2^	7.8 µM	
Impedance	Glucose	GOx‐ZnO	0.01–50 mg dL^−1^	/	0.1 mg dL^−1^	[54]
	Alcohol	AOx‐ZnO	0.01–100 mg dL^−1^	/	0.1 mg dL^−1^	
	Glucose	GOx‐ZnO	0.01–200 mg dL^−1^	/	0.1 mg dL^−1^	[55]
	Lactate	poly(3‐APBA)	3–100 mM	23 ± 3 M^−1^	1.5 mM	[56]
	Lactate	GO‐LOD	1–100 mM	/	1 mM	[57]
	Cortisol	CApt‐MBs	0.10–100 ng mL^−1^	/	2.1 pg mL^−1^	[58]
	Cortisol	Au 3D‐ nanostructured	1 pg mL^−1^‐1 µg mL^−1^	0.25 Ohm ng mL^−1^	1 pg mL^−1^	[59]
	Cortisol	Aptamer‐ZnO	1–256 ng mL^−1^	/	2 ng mL^−1^	[60]

3D: three‐dimensional; AA: ascorbic acid; AOx: alcohol oxidase; BGQDs: boron‐doped graphene quantum dots; CApt: cortisol DNA aptamer; CNTs: carbon nanotubes; DA: dopamine; E‐MIP: electro‐degraded MIP; GA: glutaraldehyde; GO: graphene oxide; GOD: glucose oxidase; ISE: ion‐selective electrode; LEG‐CS: laser‐engraved graphene‐based chemical sensor; LOD: limit of detection; LOD: lactate oxidase^[^ ^57^ ^]^; MBs: magnetic beads; MIP: molecularly‐imprinted polymers; MPA: integrating micropillar array; Ni/Cu‐MOFs: nickel‐copper metal‐organic frameworks; NCGP: Ni‐Co metal‐organic framework/Ag/reduced graphene oxide/polyurethane; Phe: phenylalanine; SilkNCT: silk fabric–derived intrinsically nitrogen (N)‐doped carbon textile; Ti_3_C_2_Tx/PB: MXene/Prussian blue; UA: uric acid; ZnO NWs: zinc‐oxide nanowires.

##### Amperometry

**Figure 2:**
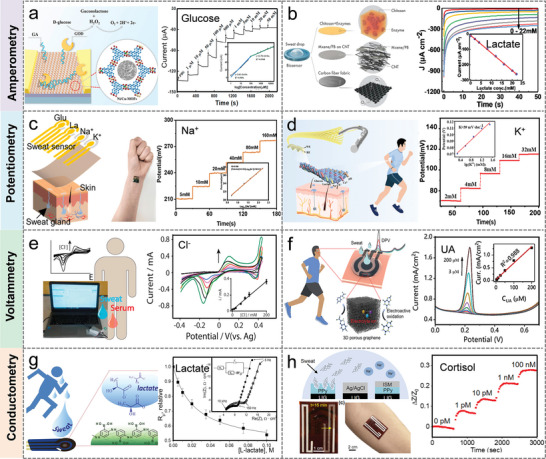
Sweat biosensors based on different electrochemical analysis methods. a) Detection mechanism and amperometry current response to glucose in field‐effect transistors based on bimetallic Ni/Cu metal‐organic frameworks. Reproduced with permission.^[^ ^43^ ^]^ Copyright 2021, Elsevier. b) A MXene‐based wearable biosensor system for lactate analysis. Reproduced with permission.^[^ ^45^ ^]^ Copyright 2019, Wiley‐VCH GmbH. c) Flexible sweat sensor systems and the potential responses for Na^+^ detection. Reproduced with permission.^[^ ^61^ ^]^ Copyright 2022, American Chemical Society. d) Schematic diagram of smart clothing based on electrochemical sensing fiber and potential response diagram toK^+^ measurement. Reproduced with permission.^[^ ^10^ ^]^ Copyright 2023, The Royal Society of Chemistry. e) Screen printed electrode for determination of Cl^−^ in sweat based on voltammetry. Reproduced with permission.^[^ ^50^ ^]^ Copyright 2018, Elsevier. f) Multichannel electrochemical sensor for UA detection based on voltammetry. Reproduced with permission.^[^ ^53^ ^]^ Copyright 2023, American Chemical Society. g) Impedance sensors for detection of lactate in sweat. Reproduced with permission.^[^ ^56^ ^]^ Copyright 2017, American Chemical Society. h) Cortisol sensor based on electrochemical impedance spectroscopy. Reproduced with permission.^[^ ^62^ ^]^ Copyright 2024, American Chemical Society.

Amperometry is the measurement of the current generated by the redox reaction of the substance to be measured in an electrolyte. When a constant potential is applied, the transfer of electrons between the electrode and the substance to be measured is proportional to the concentration of the electroactive product during oxidation or reduction of the electroactive substance. The amperometry method is ideally suited for the detection of a range of sweat metabolites because many substances to be measured are susceptible to redox reactions in the presence of inorganic and/or biological catalysts at potentials lower than those required for the electrolysis of water. **Figure** **2**a demonstrated a novel enzymatic glucose biosensor based on bimetallic Ni/Cu‐MOFs prepared via a simple, one‐step hydrothermal method.^[^ ^43^ ^]^ The performance of the sensor was obtained through a typical amperometry method, which displayed a piecewise linear relationship in the wide range (1 µM–20 mM), with a high sensitivity (26.05 µА cm^−2^ mM^−1^) and a low detection limit (0.51 µM). To address the limited detection range and sensitivity of enzyme‐based biosensors due to hypoxia in sweat. Lei et al. designed a stretchable, wearable, and modular multifunctional biosensor using MXene/Prussian blue (Ti_3_C_2_T_x_/PB) composite, which detects glucose and lactate in sweat with lasting sensitivity (Figure 2b).^[^ ^45^ ^]^ The electrochemical sensitivity for the determination of lactate was 11.4 µA mM^−1^ cm^−2^, and the detection limit was 0.67 × 10^−6^ M.

##### Potentiometry

Potentiometry allows the detection of electrolytes where the standard redox potential exceeds that of electrolyzed water. A general‐purpose potentiometer sensor consists of an ion‐selective electrode and a reference electrode. The potential of the reference electrode is independent of the composition of the sample, whereas the potential of the ion‐selective electrode follows the Nernst equation and depends on the activity of the ion to be measured so that its concentration can be determined indirectly. Wang et al. presented an ultra‐compact wearable biosensor system for monitoring biomarkers (glucose, lactate, Na^+,^ and K^+^) in sweat. The open‐circuit potential of the Na^+^ sensor showed a good linear relationship with the logarithm of the ion concentration (R^2 ^= 0.999), with a sensitivity of 43.76 mV decade^−1^ (Figure 2c).^[^ ^61^ ^]^ Potentiometric methods provide a rapid, real‐time reflection of the concentration of the substance to be measured and have been widely used for sweat ion monitoring. As shown in Figure 2d, an electrochemical sensing fiber with a special core‐sheath structure was reported.^[^ ^10^ ^]^ Based on an ion‐selective membrane, this sensing fiber demonstrated concentration variations through surface potential changes and displays a near‐Nernst behavior with sensitivities of 67, 29.4, and 39 mV decade^−1^ for Na^+^, Ca^2+^, and K^+^ sensing fibers, respectively.

##### Voltammetry

Voltammetry mode mainly includes cyclic voltammetry, differential pulse voltammetry, and square wave voltammetry, through the voltage scanning between the working electrode and the reference electrode, the peak current characteristics are extracted to determine the reactant concentration. Compared with the amperometry mode, this method could support the detection of analytes with concentrations of one part per million or even one part per trillion, which can achieve a lower detection limit. Due to its high sensitivity and stability, voltammetry has been used for the detection of Cl^−^, uric acid (UA), dopamine (DA), and ascorbic acid (AA) in sweat. Cinti et al. report a paper‐based screen‐printed electrode that utilizes the electrochemical reaction of chloride with a silver working electrode to detect Cl^−^ in sweat.^[^ ^50^ ^]^ In the electrochemical reaction, cyclic voltammetry demonstrated an anodic/cathodic peak proportional to the Cl^−^ in solution (Figure 1e). The paper sensor requires only 10 µL of samples to detect Cl^−^, and is highly correlated at concentrations up to 200 mM, with a detection limit of 1 mM. High sensitivity and low detection limits for detecting multiple biomarkers and electrolytes in sweat are essential for wearable sweat sensing. Figure 1f illustrates a multifunctional chemical sensor.^[^ ^53^ ^]^ The specific voltage waveform in differential pulse voltammetry (DPV) allows electroactive substances to be oxidized at specific potentials while hydrogen atoms and electrons are lost at the electrode, and the oxidation peak current increases with substance concentration. The sensitivity of the sensor for UA/DA/ tyrosine/AA was obtained by the DPV method to be as high as 6.49/6.87/0.94/0.16 µA µM^−1^ cm^−2^, and a low LOD was 0.28/0.26/1.43/11.3 µM.

##### Impedance

Impedance spectroscopy is a powerful tool to investigate the properties of materials and electrode reactions. In a typical three‐electrode system, the impedance of the electrode interface is obtained by applying a small alternating current signal of varying frequency at the interface between the electrode and electrolyte. The impedance method realizes the detection and analysis of biomarkers by measuring the change of impedance in the electrochemical reaction, which has the advantages of high sensitivity and low detection limit. Zaryanov et al. prepared molecularly imprinted polymers for lactate detection by electropolymerization of 3‐aminophenylboric acid (3‐APBA).^[^ ^56^ ^]^ Based on electrochemical impedance analysis, the sensor detected lactate over a range of 3–100 mM with a detection limit of 1.5 mM, and the response time was 2–3 min (Figure 1g). More importantly, the sensitivity of the sensor remains essentially unchanged over 6 months of open storage in a dry state, which is not possible with ordinary enzyme‐based devices. In addition, Garg et al. constructed a molecule‐imprinted polymer (MIP) based biochemical sensor using electrochemical impedance spectroscopy to measure cortisol levels in sweat, eliminating the need for labels or redox probes.^[^ ^62^ ^]^ The detection limit of the molecularly imprinted biosensor for cortisol concentration was as low as 1 pM, showing high sensitivity and excellent selectivity (Figure 1h).

#### 2.1.2 Optical Mode

Optical sensors utilize light conduction technology to obtain information for analysis. Due to its low cost and simplicity of operation, optical sensing is another attractive approach to developing wearable sweat biosensors. The chemical signals in sweat are converted into optical signals by chromophores or fluorophore molecules, which can be directly interpreted and read with relevant physiological information via external devices such as smartphones and portable Raman spectrometers. Currently, colorimetry, surface‐enhanced Raman scattering (SERS), fluorescence, and electrochemiluminescence based on optical techniques have been applied in the field of sweat analysis.^[^ ^63^, ^64^ ^]^

##### Colorimetry

**Figure 3:**
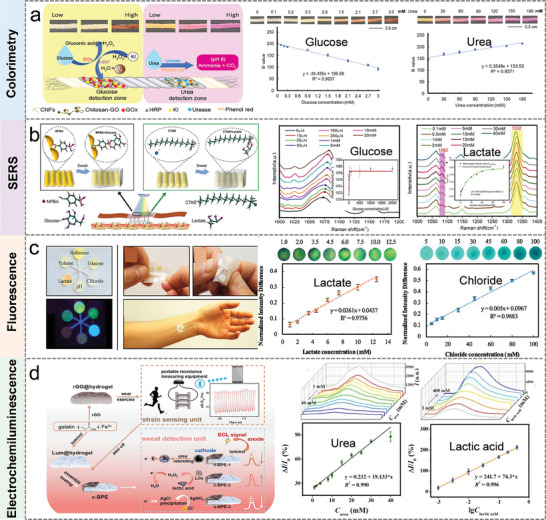
Sweat biosensors based on different optical analysis methods. a) A non‐invasive thread colorimetric wearable sensor for simultaneous detection of glucose and urea in human sweat. Reproduced with permission.^[^ ^67^ ^]^ Copyright 2020, Elsevier. b) Detection mechanism of glucose and lactate based on SERS technology. Reproduced with permission.^[^ ^64^ ^]^ Copyright 2022, Elsevier. c) Structure diagram of the flexible sweat patch and the detection performance of lactate and chloride in sweat. Reproduced with permission.^[^ ^68^ ^]^ Copyright 2020, Elsevier. d) Electrochemiluminescence sensor for urea and lactate detection. Reproduced with permission.^[^ ^69^ ^]^ Copyright 2023, Elsevier.

Colorimetry is the most widely used optical sensing technique in wearable and flexible electronics, where the sensing element undergoes a simple color change through a chemical reaction in the presence of the target analyte. This color change is commonly quantified by simple absorbance measurements, where the sensing element is irradiated and the reflected or transmitted light is recorded. Colorimetric detection of biomarkers in sweat has the advantages of simple operation, fast, low cost, strong portability, and suitable for rapid screening in the field.^[^ ^65^, ^66^ ^]^ **Figure** **3**a illustrates a modified cotton thread colorimetric sensor for simultaneous detection of glucose and urea in human sweat.^[^ ^67^ ^]^ The sensing platform has a linear range of 0.1–3 mM with a detection limit of 0.1 mM for glucose, and a linear range of 30–180 mM with a detection limit of 30 mM for urea. Due to the flexibility of the thread, this sensor was easily integrated with clothing and accessories to continuously monitor diabetes and kidney failure in real‐time through the wearer's sweat.

##### SERS

Raman spectroscopy contains abundant chemical fingerprint information at the molecular level.^[^ ^70^ ^]^ SERS with 10^6^‐10^7^‐fold enhancement in the characteristic peaks associated with chemical structures has strong sensitivity even at the single‐molecule level, and is widely used in chemical, analytical science, and biomedical research.^[^ ^70^, ^71^ ^]^ In addition, SERS signals have an extremely narrow bandwidth and could indicate the molecular fingerprint information inherent in chemical and biological systems, which is highly suitable for the detection of metabolites. Zhao et al. described a textile‐based microfluidic device that integrates SERS technology and colorimetric analysis as a multifunctional sweat sensor.^[^ ^64^ ^]^ As in Figure 3b, gold nanorods with core‐shell structure were sandwiched in a Raman reporter and deposited as SERS labels in embroidery sites for detecting target analytes in sweat. The experimental results showed that the detection range and detection limit of this sensor for glucose and lactate in sweat were 0–20 mM, (0.125 µM, S/N = 3) and 0.1‐40 mM, (0.05 mM, S/N = 3), respectively.

##### Fluorescence

The principle of fluorescent sensors is based on the phenomenon of photoluminescence. A fluorescent probe is used to label the target receptor, and when the target analyte binds to the probe, the photophysical state of the fluorophore molecule changes, resulting in a shift in the emitted fluorescent signal. The method has the characteristics of high sensitivity, excellent selectivity, and rapid reaction speed.^[^ ^63^, ^72^ ^]^ Fluorescence‐based sweat analysis is a low‐cost in vitro diagnostic and analytical method. Ardalan et al. developed a cellulose‐based wearable patch for monitoring multiple sweat biomarkers, including glucose, lactate, pH, chloride, and volume.^[^ ^68^ ^]^ The developed smart wearable sweat patch sensor consists of a fluorescent sensor probe and a microfluidic channel. As shown in Figure 3c, the detection range and detection limit (S/N = 3) of this sensor for lactate and chloride in sweat were 1.0–12.5 mM (LOD: 0.4 mM), and 10–100 mM (LOD: 5 mM), respectively.

##### Electrochemiluminescence

Electrochemiluminescence or electrogenerated chemiluminescence is a light‐emitting phenomenon on the electrode surface where luminophore species undergo an energetic electron‐transfer reaction (redox‐reaction) to form excited states and emit light. The electrochemiluminescence mechanism includes an annihilation mechanism and a co‐reactor mechanism. Electrochemiluminescence‐based detection methods do not require an external light source and have significant advantages such as ultra‐low background noise, high sensitivity, and multi‐dimensional signal extraction.^[^ ^63^, ^73^ ^]^ In Figure 3d, Hu et al. combined electrochemiluminescence with hydrogel to design a multifunctional sensing platform for monitor movement status and analyzing biomarkers in sweat.^[^ ^69^ ^]^ Sweat detection was accomplished by bipolar electrodes, where a light‐emitting tube at the anode generates an electrochemical luminescence signal in response to the analyte at the cathode. The results showed that the sensor detect urea and lactate in sweat in the ranges of 1–40 mM and 1–400 mM, respectively.

### 2.2 Nanomaterials in Sweat Sensors

Nanomaterials have a high surface area to volume ratio, excellent electron transport properties, and electrocatalytic activity, which improve the sensitivity, detection limit, and response speed of the sensor. In addition, nanomaterials also have properties such as quantum size effect, biocompatibility, easy surface modification, self‐assembly, and self‐healing capabilities. These special qualities enable sweat sensors with high sensitivity, robustness rapid time response, and long‐term stability monitoring. The emergence of the internet of things has accelerated the demand for sweat sensor yields, fueling focused nanomaterial sensor research. According to their dimensional and morphological classification, nanomaterials are mainly classified as 0D, 1D, and 2D. This section briefly introduces the key material properties of several typical nanomaterials and their application performance in sweat sensing, as shown in **Table** **2**.

**Table 2:** Key material properties and sweat sensing performance of nanomaterials.

Nanomaterial	Typical material	Material property	Analyte	Detection range	LOD	Refs.
0D	GQDs	Fluorescence activity;	UA	0–50 mM	0.99 µM	[46]
		High specific surface area	Glucose	0–0.5 mM	0.034 mM	[74]
	AuNPs	High conductivity;	Urea	0–100 mM	20 mM	[75]
		Biocompatibility	Glucose	1–10 mM	5.347 µM	[76]
	PtNPs	High conductivity;	Glucose	0–1 mM	29.15 µM	[41]
		High catalytic activity	Glucose	0.0003–2.1 mM	0.3 µM	[77]
	AgNPs	Antibacterial activity	Tyrosine	10–200 µM	3.3 µM	[78]
1D	ZnO‐NW	High surface area to volume ratio	Lactate	0–25 mM	3.61 mM	[47]
			Na^+^	0.1–100 mM	0.16 mM	
	CNTs	Conductivity;	Glucose	10 µM–5 mM	0.079 µM	[14]
		Mechanical elasticity;	Lactate	0–16 mM	3.70 mM	[27]
		3D porosity	Na^+^	10–160 mM	0.549 mM	[10]
	TiO_2_ HNT	Light‐capturing property;	Glucose	< 200 µM	0.7 µM	[79]
		Large specific surface area				
	AuNRs	Surface plasmon resonance	Glucose	0–20 mM	0.125 µM	[64]
	v‐Au NWs	Stretchability	Glucose	0–1.4 mM	10 µM	[80]
2D	GO	High conductivity;	Glucose	0.1–3 mM	0.1 mM	[67]
		Chemical stability;	Glucose	10 nM–25 mM	10 nM	[81]
		Large surface area	Glucose	0.00125–7.72 mM	1.25 µM	[82]
			UA	5–600 µM	3.7 µM	[34]
	MXene	Conductivity;	Cortisol	5–180 ng mL^−1^	0.54 ng mL^−1^	[83]
		Biocompatibility;	Glucose	0.01–3 mM	0.33 µM	[45]
		Good photothermal	Lactate	0–22 mM	0.67 µM	
	MoS_2_	Optical electronic properties;	Lactate	30 µM–5 mM	6 µM	[84]
		Large active surface	AA	10 µM–5 mM	4.2 µM	[85]

AuNRs; gold nanorods; CNTs: carbon nanotubes; GQDs: graphene quantum dots; TiO_2_ HNT: TiO_2_ hierarchical nanotubes; v‐Au NWs: vertically aligned Enokitake‐like gold nanowires; ZnO NWs: zinc‐oxide nanowires.

#### 2.2.1 0D Nanomaterials

**Figure 4:**
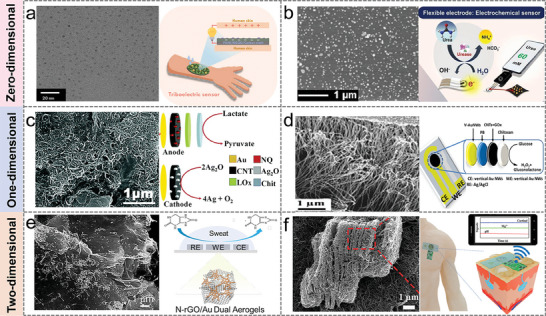
Sweat sensors are based on different materials. a) Highly conductive N‐doped graphene quantum dot‐based sweat sensor. Reproduced with permission.^[^ ^89^ ^]^ Copyright 2023, Elsevier. b) Carbonization of self‐reduced AuNPs on silk as wearable skin patches for non‐invasive sweat urea detection. Reproduced with permission.^[^ ^75^ ^]^ Copyright 2023, Elsevier. c) Stretchable e‐skin biofuel cell based on CNTs modification. Reproduced with permission.^[^ ^90^ ^]^ Copyright 2017, The Royal Society of Chemistry. d) Glucose sweat sensor based on v‐Au NWs stretchable electrode. Reproduced with permission.^[^ ^80^ ^]^ Copyright 2019, American Chemical Society. e) Dual aerogel non‐enzymatic wearable sweat sensor based on N‐rGO/Au DAs. Reproduced with permission.^[^ ^34^ ^]^ Copyright 2023, American Chemical Society. f) Sweat sensing patches based on Ti_3_C_2_T_x_, MWCNTs, and silver nanoparticles (AgNPs). Reproduced with permission.^[^ ^91^ ^]^ Copyright 2022, Wiley‐VCH GmbH.

0D nanomaterials, such as pure metals, alloys, semiconducting metal oxides, and nonmetals, have important functions in the field of sweat sensing, including molecular immobilization and labeling, reaction catalysis, and enhanced electron transfer.^[^ ^86^, ^87^, ^88^ ^]^ Graphene quantum dots (GQDs) have high fluorescence activity, low toxicity, chemical inertness, improved surface modification by π‐π conjugated surface groups, and excellent conductivity properties. Due to the large difference in electronegativity between the carbon and the dopant, doping of heteroatoms, such as N, P, B, F, and S, can significantly improve the conductivity of the sensor. In **Figure** **4**a, a novel self‐powered glucose sensor was designed based on N‐doped graphene quantum dot‐modified polyaniline nanocomposites (NGQDs/PANI) as a triboelectric layer.^[^ ^89^ ^]^ The surface charge was increased by adding electron‐rich functional groups to the NGQDs to adjust the surface electronegativity of PANI and improve the friction electric output. The results demonstrated that the sensor had a higher sensitivity for glucose detection (23.52 mM^−1^) than that of the original PANI/GOx (16.44 mM^−1^). The conductivity of the working electrode material is crucial for the sensing performance of wearable electrochemical sensors. Gold nanoparticles (AuNPs) are commonly used as working electrode materials due to their high conductivity, biocompatibility, and simple preparation process. Prophet et al. prepared flexible conductive skin patches on silk fabric using in situ deposition of AuNPs and carbonization (Figure 4b).^[^ ^75^ ^]^ This silk skin patch has excellent catalytic properties, a linear range of 0–100 mM for urea detection, and a detection limit of 20 mM, which was successfully used for urea detection in human sweat.

#### 2.2.2 1D Nanomaterials

1D nanomaterials are extensions of 0D nanomaterials in one direction with high aspect ratios and cross‐sectional diameters less than 100 nm. The mechanical elasticity of 1D nanomaterials could satisfy the key requirement for flexible electronics, namely robustness to mechanical deformation. More importantly, the charge pathway of 1D nanomaterials is limited to surface‐exposed atoms, which makes 1D nanomaterials highly sensitive to environmental perturbations and uniquely advantageous in chemical sensing applications.^[^ ^92^, ^93^ ^]^ Carbon nanotubes (CNTs) consist of one (single‐walled, SWCNTs) or several (multi‐walled, MWCNTs) concentric graphene lamellae rolled into a cylindrical shape. CNTs have excellent photoelectric, chemical, and mechanical properties, especially in electrochemical sensing. Figure 4c shows a stretchable e‐skin biofuel cell for scavenging energy from human sweat, with CNTs uniformly distributed in the anode and cathode spheres of the cell.^[^ ^90^ ^]^ This homogeneity is essential for effective electronic pathways between the active components of the anode/cathode and the underlying carbon current collecting electrodes. The cell has an open‐circuit voltage of 0.5 V and a power density close to 1.2 mW cm^−2^ at 0.2 V. Gold nanowires have the advantages of great biocompatibility, wide electrochemical window, and high electrical conductivity. It has been demonstrated that vertically aligned Enokitake‐like gold nanowires (v‐Au NWs) bonded to EcoFlex elastomers stretched to 800% strain without loss of conductivity.^[^ ^94^ ^]^ In Figure 4d, a stretchable glucose biosensor was designed based on glucose oxidase and Prussian blue nanoparticles modified vertical gold nanowire electrode.^[^ ^80^ ^]^ The sensor had a detection limit of 10 µM for glucose and a sensitivity of 23.72 µA mM^−1^ ****cm^−2^ with high selectivity.

#### 2.2.3 2D Nanomaterials

2D nanomaterials with diverse electronic properties and large specific surface areas are ideal for sensor applications. Graphene is a widely researched 2D nanomaterial that is mechanically robust and highly conductive, making it possible to create a variety of wearable sweat sensors.^[^ ^82^, ^88^, ^95^ ^]^ A dual aerogel‐based non‐enzymatic wearable sensor was proposed for the highly sensitive and selective detection of uric acid (UA) in human sweat.^[^ ^34^ ^]^ As shown in Figure 4e, the dual structural aerogels composed of Au nanowires and N‐doped graphene nanosheets (referred to as N‐rGO/Au DAs), have large active surfaces, fast electron transfer pathways, and high intrinsic activity. As an enzyme‐free wearable UA sensor, it exhibited a sensitivity as low as 3.7 µM (S/N = 3), along with good selectivity and long‐term stability. MXenes (also known as Ti_3_C_2_T_x_), with high electrical conductivity, are considered a substitute for graphene and are nanosheets exfoliated from bulk MAX chemical alloys through wet etching and other chemical methods. The peculiar structure of MXenes offers significant advantages in sensing, such as accordion‐like multilayer architectures ideal for continuous detection, and high electrical conductivity that makes sensors extremely responsive. Figure 4f demonstrated an integrated wearable sweat sensing patch for continuous analysis of stress biomarkers (e.g., cortisol, Mg^2+,^ and pH) in the resting state.^[^ ^91^ ^]^ MXenes exhibit metallic conductivity, which allows them to efficiently capture electroactive information and convert it into electrical signals. Moreover, due to its accordion‐like structure, MXenes also provides a rich set of reaction sites for analyte sensing.

### 2.3 Sweat Sensors Device

Wearable sweat‐sensing devices offer an exciting ability to monitor human physiology in real time. To be practical, wearable sensors must be comfortable, unobtrusive, and conform to the soft nature of the human body. This section analyzes recent advances in flexible skin interfaces and textiles for sweat sensors. **Table** **3** shows the advantages, disadvantages, and application scenarios of tattoo, skin patch, and textile‐based sweat sensors.

**Table 3:** Sweat sensors are based on different wear forms.

Wearing form	Advantages	Disadvantages	Application	Refs.
Tattoos	No additional equipment required;	May cause skin irritation;	Motion monitoring;	[96, 97, 98]
	Integrated microfluidic technology	Limited durability;	Disease diagnosis;	
	Direct contact with skin;	Removal difficulty;	Daily health tracking;	
	Flexible and comfortable	Limited accuracy	Health management	
Skin patches	Multifunction integration;	High manufacturing cost;	Medical monitoring;	[18, 35]
	High flexibility and good fit;	External power supply required;	Man‐machine interaction interface;	[75, 91]
	High sensitivity and selectivity	Limited durability	Personalized nutrition management	[99, 100]
Textiles	Capable of mass production;	Influence clothing comfort;	Long‐term health monitoring;	[10, 11]
	Integration with clothing;	Washing affects durability;	Military training;	[16, 37]
	High comfort;	Limited sensitivity and selectivity;	Sports equipment;	[47, 64]
	Good air permeability	Requires regular cleaning and maintenance	Sportswear	[67, 101]

#### 2.3.1 Tattoos

**Figure 5:**
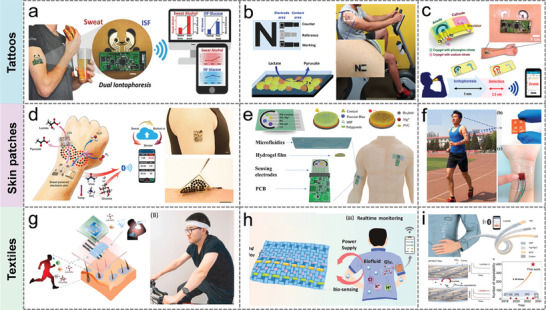
Sweat biosensors based on different wear devices. a) Wearable sweat sensor based on tattoo form.^[^ ^96^ ^]^ Copyright 2018, Wiley‐VCH GmbH. b) Tattoo biosensor for detecting lactate levels during exercise. Reproduced with permission.^[^ ^97^ ^]^ Copyright 2013, American Chemical Society. c) Tattoo‐based transdermal alcohol sensor. Reproduced with permission.^[^ ^98^ ^]^ Copyright 2016, American Chemical Society. d) Perspiration‐powered soft electronic skin for multiplexed wireless sensing. Reproduced with permission.^[^ ^35^ ^]^ Copyright 2020, The American Association for the Advancement of Science. e) Integrated wearable sweat sensing patch for mental stress analysis. Reproduced with permission.^[^ ^91^ ^]^ Copyright 2022, Wiley‐VCH GmbH. f) Non‐invasive electronic skin based on self‐powered wearable. Reproduced with permission.^[^ ^102^ ^]^ Copyright 2017, American Chemical Society. g) “Smart” headband wearable biosensor for lactate and sodium detection in sweat. Reproduced with permission.^[^ ^47^ ^]^ Copyright 2021, Elsevier. h) Multifunctional fabric biosensors for real‐time sweat monitoring. Reproduced with permission.^[^ ^103^ ^]^ Copyright 2023, Wiley‐VCH GmbH. i) Sweat biosensor based on wearable fabric. Reproduced with permission.^[^ ^11^ ^]^ Copyright 2024, Wiley‐VCH GmbH.

The electronic tattoo is as fine as hair, ultra‐thin and ultra‐soft, and can be tightly integrated with human skin for long‐term, high‐fidelity biometric sensing, making it a promising wearable sweat electronic product. As shown in **Figure** **5**a, a tattoo sensor was demonstrated for detecting glucose and ethanol in sweat.^[^ ^96^ ^]^ The sensor was easy to use and mechanically stable during physical manipulation when applied to the upper arm of the body. When the tattoo sensor was combined with a flexible electronic board and similar (twisting and bending) strains were applied, the sensor did not cause significant structural damage, reflecting the conformal and flexible properties of the entire integrated tattoo device. The work of Figure 5b depicted real‐time noninvasive lactate monitoring of human sweat using a flexible tattoo electrochemical biosensor.^[^ ^97^ ^]^ This new skin wear enzyme biosensor exhibited chemical selectivity toward lactate with linearity up to 20 mM, and was resilient to continuous mechanical deformation from epidermal wear. In contrast to traditional lactate blood draws, tattoo patch‐based epidermal biosensors are non‐invasive, simple to operate, and cause no hindrance to the wearer. Figure 5c illustrates a wearable temporary tattoo biosensing system of real‐time noninvasive alcohol monitoring by integrating printed and flexible iontophoretic sensing electrodes with wireless electronics.^[^ ^98^ ^]^ This novel skin‐worn, low‐cost, non‐invasive alcohol monitoring device measures alcohol levels in sweat in real‐time, avoiding lengthy and expensive procedures. To enable real‐time alcohol monitoring with wearable sensors, the tattoo alcohol sensor integrates flexible printed electronic circuitry that wirelessly controls the entire ionic current sensing operation and transmits the data to a laptop or mobile device via Bluetooth communication.

#### 2.3.2 Skin Patches

Electronic skin has mechanical durable features like human skin. Moreover, the compatibility of soft e‐skin enables wearable devices to sense biochemicals from the environment and the human body, showing great potential for wearable personalized health monitoring at the molecular level. Yu et al. reported an integrated perspiration‐powered integrated electronic skin that uses untreated human sweat as a biofuel to provide a power supply for the device (Figure 5d).^[^ ^35^ ^]^ The platform is ultra‐thin and transparent, utilizes low‐power electronics, and integrates the electronics onto a soft substrate, presenting a fully compatible e‐skin with maximum comfort and wearability. It selectively monitors key metabolic analytes (e.g., urea, NH_4_^+^, glucose, and pH) and skin temperature during prolonged physical activity and wirelessly transmits the data to a user interface via Bluetooth. In Figure 5e, the combination of a microfluidic chip for sweat collection, a high‐sensitivity sensing platform for biomarker detection, and in situ signal processing circuits for signal transduction, conditioning, and wireless transmission could constitute an integrated wearable sweat sensing patch.^[^ ^91^ ^]^ Similarly, the introduction of a self‐powered piezoelectric wearable device into the skin patch enables noninvasive detection of lactate, glucose, uric acid, and urea in sweat without the need for an external power source or battery (Figure 5f).^[^ ^102^ ^]^

#### 2.3.3 Textiles

Electronic textile biosensors, which are realized by integrating electronic components and functionality into a textile substrate, have emerged as attractive wearable devices for human biosensing. Due to its textile nature, it offers tight and friendly skin contact and potential integration with real clothing, which is interesting for analyzing the composition of sweat on the skin during the user's daily activities. In recent years, there have been various e‐textile‐based wearable platforms for human health monitoring. Zhao et al. developed a fully integrated wearable sweat testing platform based on a thread‐based biosensor for accurate lactate and sodium detection in human sweat during exercise (Figure 5g).^[^ ^47^ ^]^ The constructed “smart” head‐mounted device integrates biosensors and their circuitry for signal reading and transmission, which can communicate wirelessly with a smartphone to transmit data. Due to considerable flexibility, smart fibers with on‐demand functionality are ideal candidates for the fabrication of noninvasive and conformal bioelectronic devices. As shown in Figure 5h, Tong et al. reported a multifunctional textile patch based on reduced graphene oxide (rGO)/tetraphenylamine (TANi) fibers for the simultaneous monitoring of biomarkers and energy supply.^[^ ^103^ ^]^ Weaving multiplexed sensing fabric into a single piece of gauze and integrating it with a circuit board enables wearable continuous monitoring, storage, and even data communication. In Figure 5i, a scalable fabric biosensor was demonstrated for long‐term repeatable detection of lactate in sweat.^[^ ^11^ ^]^ The fabric biosensor creates a stable interface between the molecularly imprinted polymer and the fiber electrode, allowing for long‐term repeated detection of lactate more than 400 times. A fabric biosensor with a length of 20 m and width of 0.5 m was demonstrated using industrial sewing techniques, showing its potential for large‐scale applications.

## 3 Wearable Energy Harvesters

Rapid advances in bioelectronics are driving the development of wearable sweat biosensors for monitoring body movement and personalized healthcare. Currently, one of the challenges for wearable sweat biosensors is to ensure sustained use with a reliable power supply. Unlike conventional rigid batteries, which need to be replaced or recharged intermittently, energy harvesters offer a battery‐free way to efficiently harvest energy from human movement and the environment. The energy harvester could convert biomechanical energy, biochemical energy, thermal energy, and solar energy into electrical energy, considerably improving the feasibility and convenience of wearable sweat biosensors for continuous health monitoring. Depending on the energy source, energy harvesters are categorized into triboelectric nanogenerators, piezoelectric nanogenerators, thermoelectric generators, biofuel cells, and photovoltaic cells.^[^ ^104^, ^105^ ^]^ In this section, various types of energy harvesters are described in detail, covering their working mechanisms, structural components, advantages, and disadvantages.

### 3.1 Triboelectric Nanogenerators

**Figure 6:**
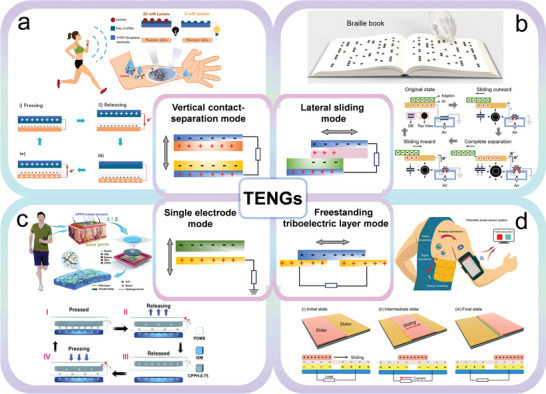
Working modes and applications of TENGs: a) Vertical contact‐separation mode; Self‐powered molecular imprinted polymers‐based triboelectric sensor for noninvasive monitoring lactate. Reproduced with permission.^[^ ^17^ ^]^ Copyright 2022, Elsevier. b) Lateral sliding mode; Refreshable braille display system based on triboelectric nanogenerator and dielectric elastomer. Reproduced with permission.^[^ ^109^ ^]^ Copyright 2020, Wiley‐VCH GmbH. c) Single‐electrode mode; Stretchable triboelectric self‐powered sweat sensor fabricated from self‐healing nanocellulose hydrogels. Reproduced with permission.^[^ ^20^ ^]^ Copyright 2022, Wiley‐VCH GmbH. d) Freestanding triboelectric layer mode; Wireless battery‐free wearable sweat sensor powered by human motion. Reproduced with permission.^[^ ^36^ ^]^ Copyright 2020, The American Association for the Advancement of Science.

Since its first report in 2012, triboelectric nanogenerators (TENGs) have drawn extensive attention as a typical energy harvesting technology.^[^ ^106^ ^]^ The triboelectric effect is a contact electrification phenomenon in which certain materials become electrically charged when they are separated from another material with which they were in contact. Researchers have investigated the polarity of friction materials based on their ability to gain electrons (electronegativity) or lose them (electropositivity) when they come into contact with other materials. According to the configuration and driving mode, 4 operational modes were proposed to collect different forms of mechanical energy, including vertical contact‐separation mode,^[^ ^17^ ^]^ lateral sliding mode,^[^ ^101^ ^]^ single electrode mode,^[^ ^20^ ^]^ and freestanding triboelectric layer mode^[^ ^36^ ^]^ (**Figure** **6**). As an emerging energy harvesting device, the TENGs convert biomechanical energy (body motion, joint motion and mechanical vibration) into electrical energy, which is a hot topic in the field of energy supply for wearable sweat sensing.^[^ ^107^, ^108^ ^]^

Vertical contact‐separation mode is the basic mode of TENGs, where charge transfer is generated by the contact and separation of 2 different materials, creating a potential difference and thus generating electrical energy. This mode of TENGs has a simple structure and can generate periodic alternating current output, making it suitable for harvesting energy from human movement and developing pressure sensors. Based on the vertical contact‐separation mode, Figure 6a depicted a self‐powered molecular imprinted polymer‐based triboelectric sensor (MIP‐TES) for non‐invasive monitoring of lactate levels in human sweat.^[^ ^17^ ^]^ MIP‐modified lactate sensors were applied to the triboelectric electric nanogenerators that convert mechanical energy acquired in contact and separation into electrical energy output. MIP‐TES transfer charge at contact through triboelectric effect and establish potential difference upon separation. Subsequently, the electrons were reversely driven by electrostatic induction under the action of external afterforce to reduce the induced charge, and the generation and regeneration of voltage and current signals were realized. The TENGs in lateral sliding mode generate continuous electrical energy output by sliding 2 friction materials in parallel directions under the action of external forces. Its energy conversion efficiency is higher than that of TENGs in vertical contact‐separation mode, and it can produce high‐frequency continuous electrical output. Qu's group has designed a secure dielectric elastomer braille device powered by a friction electric nanogenerator, promising the development of an updatable, flexible, and portable braille book (Figure 6b).^[^ ^109^ ^]^ In the sliding mode, the TENGs transfer charge between the Kapton film and the aluminum foil through the triboelectric effect, creating a potential difference. The resulting electrostatic attraction was used to stretch the dielectric elastic film to form raised braille points. TENGs generated an output voltage of up to 3.25 kV, enough to deform the dielectric elastomer membrane, while generating a current of only 2 µA, making it safe for humans.

The single electrode mode TENGs evolved from the contact‐separation mode and in‐plane sliding mode. The freely moving triboelectric layer is in contact with the fixed electrode to generate an electric charge, which produces electrical energy during contact and separation. The simplicity of this mode structure increases flexibility and utility in self‐powered sensors and energy harvesting applications. Figure 6c demonstrates a self‐powered sweat biosensor that detects ions in real time via the friction electric effect.^[^ ^20^ ^]^ The self‐powered device operates through a TENG single‐electrode operating mechanism, with ion‐selective membrane (ISM) acting as a positive friction electric material and PDMS as a negative friction electric material. Due to biomechanical vibrations, ISM and PDMS simultaneously undergo periodic contact separation cycles, generating unique electrical signals. The self‐powered sensor has excellent flexibility and high sensitivity, which can meet the needs of real‐time and continuous monitoring of electrolyte concentration in sweat during exercise. The free‐standing triboelectric layer model is developed based on the single‐electrode model, which not only has higher energy conversion efficiency but also works under noncontact conditions, greatly reducing material wear and energy consumption. In Figure 6d, a wearable platform based on a free‐standing triboelectric nanogenerator (FTENG) was proposed to efficiently extract energy from body motion.^[^ ^36^ ^]^ The working mechanism of FTENG was explained as a coupled effect of contact energization and in‐plane sliding charge transfer. In the initial state, the grating slider completely overlaps one stator electrode and there is no charge flow between the stator electrodes due to electrostatic equilibrium. The unidirectional sliding process results in charge flow between the stator electrodes until the grating slider completely overlaps a second stator electrode with opposite polarity. The well‐designed FTENG displayed a high‐power output of 416 mW m^−2^. Through seamless system integration and efficient power management, a battery‐free triboelectric drive system was demonstrated to power a multi‐channel sweat sensor.

### 3.2 Piezoelectric Nanogenerators

**Figure 7:**
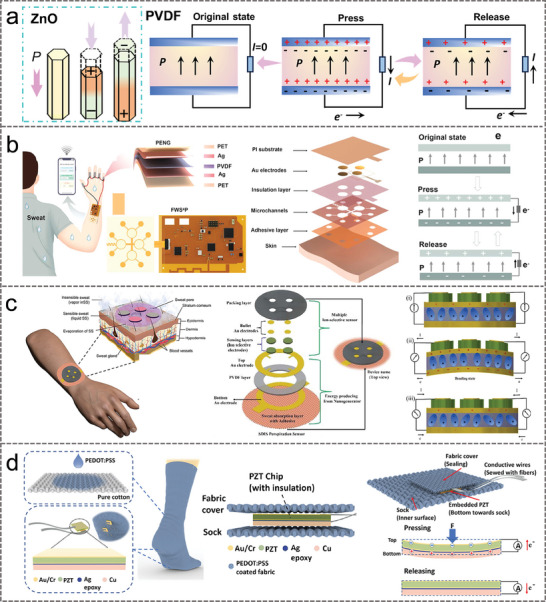
a) Structure and working mechanism of PENGs. b) PENGs convert biomechanical energy from freely movable joints into electricity. Reproduced with permission.^[^ ^23^ ^]^ Copyright 2022, Elsevier. c) Mechano‐driven multi‐ion sensor based on piezo‐ionophoretic coupling for sweat monitoring. Reproduced with permission.^[^ ^115^ ^]^ Copyright 2022, Elsevier. d) Working principle of PZT chip and integration strategy for S^2^‐sock. Reproduced with permission.^[^ ^116^ ^]^ Copyright 2019, American Chemical Society.

In 2006, Professor Zhonglin Wang et al. successfully developed a nanogenerator for the first time in the world by using piezoelectric zinc oxide (ZnO) nanowire arrays to convert mechanical energy into electrical energy, opening a new chapter in nanoscience and technology.^[^ ^110^ ^]^ Taking the N‐type ZnO structure as an example, under normal conditions, Zn^2+^ and O^2−^ are tetrahedrally stacked along the C‐axis with their centers overlapping each other (**Figure** **7**a). When the compression stress is applied to the material, the charge center would be dislocated, generating a dipole moment and inducing the generation of piezoelectric potential.^[^ ^111^ ^]^ In addition, the conversion of mechanical to electrical energy can also be achieved by utilizing the piezoelectric properties of polyvinylidene fluoride (PVDF) and barium titanate (BaTiO_3_) materials (Figure 7a).^[^ ^112^, ^113^, ^114^ ^]^ The piezoelectric nanogenerator (PENGs) structure typically includes a top and bottom electrode that collects the charge generated by the piezoelectric effect. The process of applying and releasing pressure is reversible each time, and PENGs repeat the charge separation and recovery process to achieve the cyclic conversion of energy.

The PENGs have the features of low power consumption, simple design, flexibility, and excellent mechanical stability.^[^ ^117^, ^118^ ^]^ Figure 7b illustrates a battery‐free sweat sensing system, where PENGs efficiently convert biomechanical energy from freely movable joints (finger, cubital fossa, and popliteal space) into electricity serving as the self‐powering module.^[^ ^23^ ^]^ PENGs utilize their piezoelectric effect to generate periodic changes in charge density through the periodic bending and recovery of PVDF, thereby forming a current between the upper and lower electrodes and realizing energy harvesting. In the practical scenario application, the piezoelectric properties of the PENG were utilized to convert the mechanical motion of the joints into electrical energy to power the sweat analysis, and the sensing information was transmitted wirelessly to the user interface via a flexible integrated circuit and Bluetooth module. To address the problem of lack of materials and unreliable power supply methods in the field of wearable sensing, a fully flexible Mechano‐Driven Ion‐Selective (MDIS) perspiration sensor was proposed.^[^ ^115^ ^]^ As illustrated in Figure 7c, in the initial stage, the dipoles were randomly oriented and the net electrical output response was zero. When the device was bent, the dipoles were aligned in the PVDF layer and an effective output response was observed. Positive and negative charges were separated and accumulated near the gold electrodes and collected by the ionophore cross‐linked polyaniline to produce a positive electrical output. When the applied stress is released, the dipoles return to their relaxed state, producing a negative electrical output. The prepared PVDF‐based piezoelectric nanogenerator attached to a medical tape, gives an electrical output of ≈2.5 V and ≈10 nA under bending conditions.

Wearable devices that rely on hybrid mechanisms have the advantage of creating smarter systems for healthcare, motion monitoring, and smart home applications. Zhu et al. developed a self‐powered and self‐functional sock (S^2^‐sock) by hybrid integration of friction electro‐nanogenerator (TENG) with zirconate titanate (PZT) piezoelectric sensor for realize diversified functions including energy harvesting and sensing various physiological signals (Figure 7d).^[^ ^116^ ^]^ Benefitting from the high sensitivity to mechanical forces, the PZT sensor embedded in the sock enables precise and efficient pressure sensing. The device collected an output power of 1.71 mW with a mild jump at 2 Hz and a load resistance of 59.7 MΩ. Based on the sensor fusion concept, the outputs of the TENG and PZT sensors are effectively fused together under motor activity to quickly detect sweat levels.

### 3.3 Thermoelectric Generators

**Figure 8:**
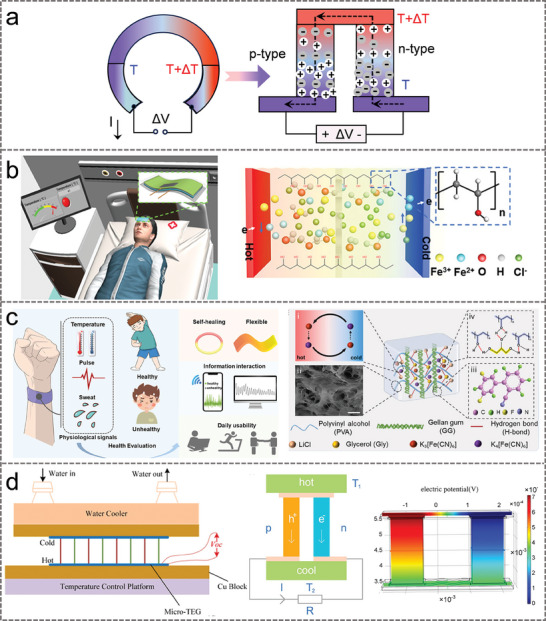
a) Structure and working mechanism of TEGs. b) A gel electrolyte‐based thermogalvanic generator for body temperature monitoring. Reproduced with permission.^[^ ^119^ ^]^ Copyright 2021, American Chemical Society. c) The working mechanism of the gel with the redox couple Fe(CN)_6_^3–/4–^ based on the thermogalvanic effect. Reproduced with permission.^[^ ^99^ ^]^ Copyright 2024, American Chemical Society. d) Test the device and working principle of a single thermocouple. Reproduced with permission.^[^ ^120^ ^]^ Copyright 2019, American Chemical Society.

The operating principle of thermoelectric generators (TEGs) is based on the thermoelectric effect, whereby an electric current is generated by a voltage difference caused by a temperature difference. As shown in **Figure** **8**a, when there was a temperature difference (Δ*T*, *T* is the absolute temperature) between the 2 ends of a conductor, the electrons and holes within the conductor would migrate due to the difference in free energy, thus creating a potential difference between the 2 ends of the conductor. This potential difference is connected by an external circuit and generates a current, which is eventually converted into electrical energy output. Depending on the major charge carriers, thermoelectric materials are categorized as *n*‐type (electron carriers) and *p*‐type (hole carriers). The overall output performance of the thermoelectric nanogenerator is improved by connecting multiple pairs of *n‐*type and *p*‐type thermoelectric materials in series.

TEGs convert thermal energy directly into electrical energy, realizing effective utilization and conversion of energy with high efficiency, and are commonly used for thermal energy capture and passive sensing.^[^ ^121^, ^122^ ^]^ To avoid the frangibility and complex preparation of traditional thermoelectric materials, a gel electrolyte‐based thermogalvanic generator with Fe^3+^/Fe^2+^ as a redox pair was fabricated.^[^ ^119^ ^]^ As shown in Figure 8b, the working mechanism of the device was based on the implantation of a polyvinylidene fluoride diaphragm with microporous distribution in the gel, which forms a thermal barrier between the 2 halves of the gel and effectively improves the Seebeck coefficient by reducing the thermal conductivity. TEGs could be used not only for thermal energy harvesting but also have the potential to serve as highly sensitive and stable self‐powered multimodal electronic skins. Tian et al. proposed a passive multimodal electronic skin for real‐time human health assessment based on a thermoelectric hydrogel.^[^ ^99^ ^]^ The hydrogel network consists of poly(vinyl alcohol)/low acyl gellan gum with Fe(CN)_6_^3–/4–^ as the redox couple (Figure 8c). When this thermoelectric hydrogel was in a temperature gradient, a stable temperature difference was formed between the 2 ends, which utilized the ambient thermal energy to generate thermoelectricity. The results showed that the device had a thermal power of 2.04 mV K^−1^, rapid self‐healing in less than 10 min, and a conductivity of 2.56 S m^−1^. In order to increase the voltage and power density of the TEGs, a flexible wearable thermoelectric generator made of Bi_2_Te was proposed.^[^ ^120^ ^]^ A thermocouple was formed by joining one side of *p*‐type and *n*‐type thermoelectric materials based on the Seebeck effect of thermoelectric materials (as shown in Figure 8d). When the temperature difference reaches 50 K, the output voltage of the TEGs would be no less than 520 mV, and the power density reach 11.14 mW** **cm^−2^.

### 3.4 Biofuel Cells

**Figure 9:**
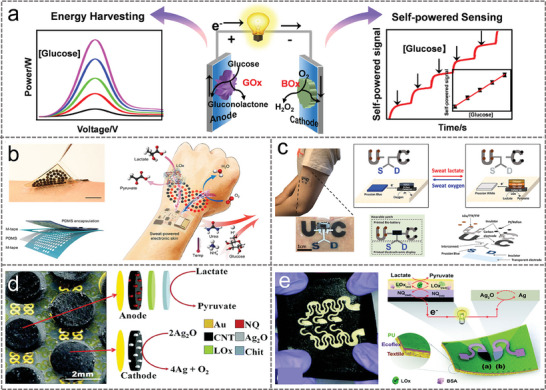
a) Conceptual illustration of glucose BFCs, showing the key working principle and applications for energy harvesting and self‐powered sensing. b) Perspiration‐powered soft electronic skin (e‐skin) based BFC for multiplexed wireless sensing. Reproduced with permission.^[^ ^35^ ^]^ Copyright 2020, The American Association for the Advancement of Science. c) Bio‐battery integrated electrochromic patch. Reproduced with permission.^[^ ^27^ ^]^ Copyright 2022, Elsevier. d) Electronic skin‐based biofuel cells for scavenging energy from human sweat. Reproduced with permission.^[^ ^90^ ^]^ Copyright 2017, The Royal Society of Chemistry. e) Composition and redox reactions of stretchable lactate BFCs. Reproduced with permission.^[^ ^37^ ^]^ Copyright 2016, The Royal Society of Chemistry.

Biofuel cells (BFCs) convert chemical energy in biological liquids (such as blood, sweat, tears, saliva, etc.) directly into electricity with the aid of biocatalysts.^[^ ^123^, ^124^ ^]^ **Figure** **9**a illustrated a typical schematic of BFCs based on enzymatic glucose reaction, where the glucose oxidase (GOx) enzyme was immobilized at the anode electrode to facilitate the glucose oxidation reaction to produce protons and electrons. At the cathode, bilirubin oxidase (BOx) enzyme was immobilized for the reduction of oxygen to water. These bioelectrochemical reactions are utilized at the electrode surface to generate current and electricity. On one hand, the current intensity and corresponding electrical energy involved in these redox reactions are directly proportional to the fuel concentration. BFCs can be used to power biosensors or charge batteries and supercapacitors (Figure 9a, left). On the other hand, BFCs can serve as self‐powered biosensors for measuring concentrations of fuels (target analytes), inhibitors or activators of reactions (Figure 9a, right). Due to uncontrollable issues with wearable devices, including the instability of enzymes, the unexpected dynamic changes in the corresponding biological fluids and the surrounding environment (e.g., changes in temperature, pH, humidity, or oxygen levels), as well as the flexibility, stability, and overall mechanical elasticity of the system under severe strain. Thus, there are still challenges to the success of wearable BFCs.

Unlike other energy harvesters, BFCs use fuels such as glucose and lactate that are both sustainable and consistent in human fluids, tears, sweat, and urine and have broad applications in the field of personalized medicine. Yu et al presented a biofuel‐powered soft electronic skin for multiplexed metabolic sensing (Figure 9b).^[^ ^35^ ^]^ The battery‐free e‐skin contains multimodal sensors and a highly efficient lactate biofuel cell to achieve high power intensity and long‐term stability. In untreated human sweat, the biofuel cell exhibited the maximum power density of 3.5 mW cm^−2^, and demonstrated good stable performance over 60 h of continuous operation. It selectively monitors key metabolic analytes (e.g., urea, NH_4_^+^, glucose, and pH) and skin temperature during prolonged physical activity and wirelessly transmits the data to a user interface via Bluetooth. Wearable BFCs were widely acclaimed as self‐powered sensors for non‐invasive monitoring of human physiological status. However, the energy generated by such devices is often insufficient to power the accompanying measurement readout systems and communication protocols. For this reason, Hartel et al. proposed a reversible fully printed electrochromic self‐powered sensor (ESPS) for on‐body sweat lactate monitoring.^[^ ^27^ ^]^ The device was realized by integrating a lactate‐oxidizable BFC anode, an oxygen‐reducible BFC cathode, and a reversible Prussian blue (PB) electrochromic display on a single wearable patch, as depicted in Figure 9c. Lactate and oxygen‐sensing electrodes were combined with a PB display to visualize the sensed information. The wearable, resettable electrochromic biosensor monitors sweat biomarkers and is fully sweat‐powered. The BFC generated power up to 13 µW scm^−2^, while bleaching the electrochromic display in 2 min requires only half that power, demonstrating efficient performance.

The development of BFCs is limited by low power density and the lack of soft, stretchable properties that are essential to achieve skin‐contact wearable biofuel systems. A soft, deterministically stretchable electronic skin‐based biofuel system (E‐BFC) was described with a power density of ≈1.2 mW cm^−2^ at an open circuit voltage of 0.2 V (see Figure 9d).^[^ ^90^ ^]^ The high‐power density was achieved through a unique combination of photolithographically patterned scalable electronic frameworks with screen‐printed, densely arranged arrays of 3D carbon nanotube bioanodes and cathodes in a scalable “island bridge” configuration. The E‐BFC maintains its performance even at 50% repetitive strain and remains stable for 2 days. When applied directly to the human skin, the E‐BFC generates ≈1 mW during motion. In the self‐powered sweat biosensor, BFCs could not only be used as a chemically driven energy harvester to provide a continuous and stable power supply for the sensor but also as an active sensor to achieve accurate detection of key biomarkers in sweat (such as lactic and glucose). In Figure 9e, glucose and lactate BFCs with single‐enzyme and membrane‐free configurations generated the maximum power densities of 160 and 250 µW cm^−2^ with open circuit voltages of 0.44 and 0.46 V, respectively.^[^ ^37^ ^]^ By providing power signals proportional to the sweat fuel concentration (lactate, glucose), these stretchable devices act as highly selective and stable self‐powered textile sensors.

### 3.5 Photovoltaic Cells

**Figure 10:**
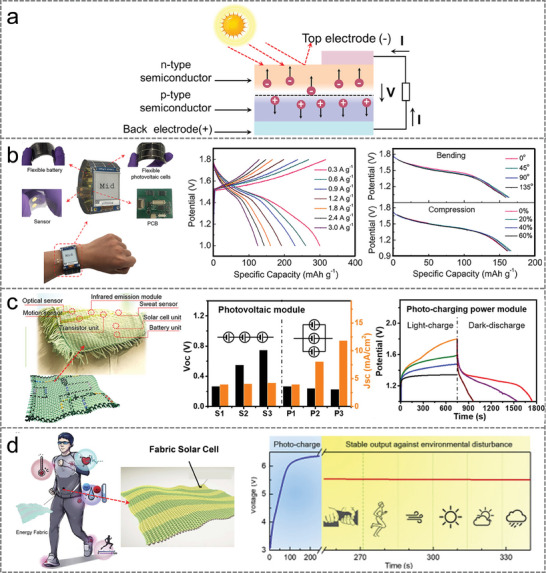
a) Structure and mechanism diagram of PVs. b) Self‐powered smartwatch for continuous sweat glucose monitoring. Reproduced with permission.^[^ ^28^ ^]^ Copyright 2019, American Chemical Society. c) Non‐printed circuit textiles based on solar cells for sweat sensing. Reproduced with permission.^[^ ^30^ ^]^ Copyright 2021, Springer Nature. d) Wearable bioelectronic devices based on optoelectronic energy harvesters. Reproduced with permission.^[^ ^125^ ^]^ Copyright 2020, Elsevier.

Photovoltaic cells (PVs) utilize the photovoltaic effect of semiconductors to directly convert the light energy of sunlight into electrical energy, which is a renewable and pollution‐free energy harvester. As shown in **Figure** **10**a, the basic structure of a PVs is a large area of planar P‐N junction. PVs are generally made of semiconductor materials such as silicon, in which phosphorus and boron atoms are doped to form *p*‐type and *n*‐type semiconductors, respectively. When the sunlight hits the P‐N junction, the P‐N junction absorbs the light energy and excited electrons and holes, generating an electric current when connected to an external circuit.

With the continuous advancement of wearable technology, researchers have also focused on converting photovoltaic energy into electrical energy to power wearable sweat biosensors. However, traditional PVs primarily rely on rigid crystalline silicon, which poses challenges for achieving device flexibility and stretchability. Therefore, there is an urgent need for flexible PVs as an efficient energy harvester to match the long‐term, continuous energy requirements of sweat biosensors. A self‐powered and fully integrated smartwatch with flexible PVs acting as an energy device has reportedly been successfully fabricated^[^ ^28^ ^]^ (Figure 10b). The PVs and the rechargeable battery form the “strap”, and the electrochemical glucose sensor, customized circuitry, and display unit form the “dial”. The as‐fabricated batteries yield the highest capacity of 301 mA h g^−1^ at 0.3 A g^−1^. Besides, the battery retains 91.86% of the initial capacitance after 1000 charging/discharging cycles at a high current density of 1.8 A g^−1^. Recently, energy and sensor devices in the shape of fibers or fabrics have gained wide attention. In Figure 10c, a nonprinted integrated circuit textile by weaving method was reported with wireless monitoring and logic computing capabilities for continuous body artificial intelligence monitoring.^[^ ^30^ ^]^ The device was powered by a textile‐based module that utilizes solar energy collection and storage to uninterruptedly convert information about body movement, sweating, and ambient light into electrical signals through 3 sets of textile‐based sensors: sweat, optical, and motion. A complete photovoltaic charging module can reach 1.8 V in sunlight, allowing for 18 min of continuous 0.1 mA power output. This single piece of fabric requires no additional external power or signal cable connections, is soft and pleasant, and allows continuous monitoring of daily healthcare tasks around the clock.

With the prominent progress in wearable bioelectronics, body‐area sensor networks with communication and electronic system integration enable effective and accurate physiological monitoring and treatment. A photo‐rechargeable fabric as a high‐performance and sustainable power source for human sensor networks was proposed (Figure 10d).^[^ ^125^ ^]^ The fabric consists of a photovoltaic energy harvesting component and a rechargeable fabric cell to form a self‐charging power unit. The fabric could be charged up to 6.4 V under ambient solar irradiation and continue to provide electric power at a discharging current of 0.1 mA for 2 h. In addition, light‐charged fabrics on the body can be used to drive a network of body area sensors comprising temperature sensors, body motion sensors, and humidity sensors.

## 4 Energy Management of Self‐Powered Sweat Sensors

Energy harvesters have the capability to convert biomechanical, biochemical, thermal, and solar energy into electrical energy, providing the necessary power support for sweat sensing and wearable electronics. However, self‐powered devices cannot directly power conventional microelectronic devices due to irregular, unstable and inefficient power output. Therefore, self‐powered sweat sensors generally require rational circuit designs to best utilize the collected power to provide a stable power supply for wearable electronic devices. Microcontrollers (MCUs) play a central control and processing role in a circuit or power system. Advanced sensing systems use MCUs to control and coordinate the different modules in the system, including sensors, power supplies, data processing, and communications. This section focuses on various types of self‐powered technologies for power management, sensing, data processing, and wireless communications, and presents the latest advances in low‐power strategies.

### 4.1 Power Management Integrated Circuit

Currently, most electronic devices and units used in wearable sensing systems are driven by direct current (DC) power supplies with voltages of a few volts. Depending on the type of power supply and power consumption requirements, power regulation and management modules typically include rectifiers, DC/DC converters, and capacitors/batteries. BFCs, PVs, and TEGs energy output currents are normally DC and could directly charge energy storage units.^[^ ^90^ ^]^ On the other hand, energy harvesters such as TENGs and PENGs require rectification due to their alternating current (AC) characteristics.^[^ ^23^, ^36^ ^]^ High output performance is easier to achieve with a DC power supply than with an AC signal‐based energy harvester. In the whole operation mode of the self‐powered sensing system, the overall power consumption could be reduced by improving energy utilization efficiency and reasonable power management strategies. The requirements and differences in power management of different energy harvesters will be systematically analyzed.

#### 4.1.1 Triboelectric Nanogenerators

TENGs convert mechanical energy into electrical energy through contact initiation and electrostatic induction and have the characteristics of high output voltage, low current, and large matching loads.^[^ ^126^ ^]^ The output voltage of TENG can be reduced by means of an inductive transformer, capacitive transformer, and LC oscillating circuit. The energy generated by TENGs is usually rectified by full‐wave rectifiers and then stored in capacitors or batteries. TENGs generate pulsed energy output, which can be integrated with energy storage units such as supercapacitors, and lithium batteries to provide a long‐term stable energy supply.

#### 4.1.2 Piezoelectric Nanogenerators

PENGs convert mechanical vibrations into electrical energy based on the piezoelectric effect, and the output voltage is commonly an AC signal that needs to be converted into a DC voltage for use through rectification and filtering processing.^[^ ^127^ ^]^ The output power of PENGs depends on the frequency of the excitation (the frequency of the mechanical vibration) and the amplitude (the strength of the vibration). The power management unit needs to integrate the maximum power point tracking algorithm to guarantee that PENGs are always operating in the most efficient state. To ensure a continuous and stable power supply, it is necessary to integrate the PENGs with an energy storage device, such as a supercapacitor or a battery. PENGs are applied to capture mechanical vibration energy of a wide range of frequencies and are suitable for human motion energy collection.

#### 4.1.3 Thermoelectric Generators

TEGs utilize temperature differences to produce electrical energy and have a low voltage and relatively high current output. The output voltage of TEGs after collecting body heat energy is generally about tens of millivolts, which needs to be boosted (with the help of an ultra‐low voltage booster or a DC‐DC converter) to satisfy the power supply voltage requirements of the device or sensor. In practical applications, TEGs need to optimize load matching to achieve high working power density. The technique of dynamically changing the switching frequency of the DC/DC converter is used to adjust the input impedance of the booster to improve the energy conversion efficiency of the device. The output power of TEGs fluctuates with temperature differences, and energy buffering mechanisms, such as supercapacitors or batteries, need to be integrated into the circuit system design to smooth the output and provide a stable power supply.

#### 4.1.4 Biofuel Cells

BFCs are an energy‐harvesting device that converts chemical energy into electrical energy, and its output performance depends on a specific biochemical environment or substrate.^[^ ^128^ ^]^ Unlike high‐power‐density PVs, BFCs provide low‐power, stable, but potentially decaying electrical output over time. Given the low output voltage of BFCs, it is necessary for their power management units to possess a boost function, enabling them to meet the voltage requirements of electronic devices. To prevent voltage inversion and electrochemical degradation from damaging the BFCs, the power management design also required to be equipped with protection mechanisms, such as integrating voltage monitoring and disconnection functions in the power management system. In addition, BFCs produce erratic or intermittent electrical energy output and require integrated energy storage systems (such as supercapacitors, and batteries) to ensure the proper operation of the device.

#### 4.1.5 Photovoltaic Cells

PVs are one of the sustainable power sources that utilize solar energy and convert it into electrical energy.^[^ ^129^ ^]^ PVs have a low output voltage, and normally DC‐DC converters (e.g., boost converters) are used to increase the voltage to match the voltage demand of the battery or load. In addition, the power output of PVs is directly affected by the intensity of solar light. Therefore, it is essential to use a suitable energy storage device (capacitor or battery) to store the energy obtained from the sunlight in the energy buffer so that the device can work properly even in the case of low or no light. The circuit design based on the photovoltaic cell energy harvesters should also ensure that the load can operate stably under different energy supply conditions. Compared with other energy collectors, the energy management unit of PVs focuses more on the maximum power point tracking algorithm and energy storage management.

### 4.2 Signal Conditioning and Processing

**Figure 11:**
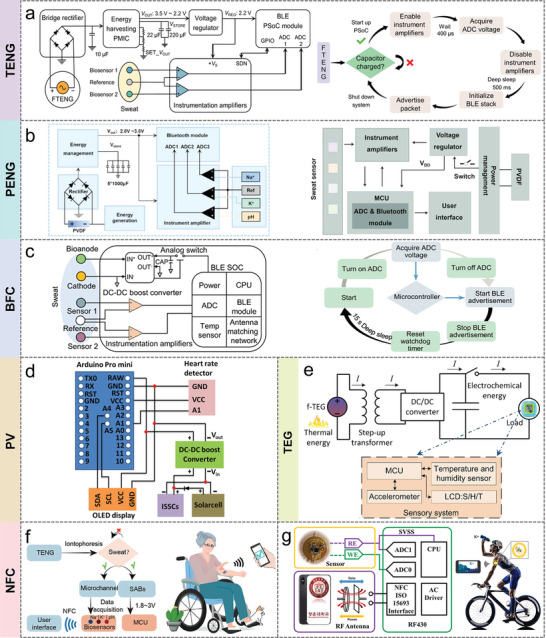
a–e) Energy management strategies for different types of energy harvesters. (a) Circuit design for signal acquisition and data processing based on TENG mode. Reproduced with permission.^[^ ^36^ ^]^ Copyright 2020, The American Association for the Advancement of Science. (b) Logic block diagram of power management, signal processing, and user interface wireless transmission based on PENG mode. Reproduced with permission.^[^ ^23^ ^]^ Copyright 2022, Elsevier. (c) Energy management and signal processing logic diagram based on BFC self‐powered system. Reproduced with permission.^[^ ^35^ ^]^ Copyright 2020, The American Association for the Advancement of Science. (d) Pulse sensor based on ISSCs, solar cell, and DC‐DC boost converter assembly. Reproduced with permission.^[^ ^130^ ^]^ Copyright 2019, Elsevier. (e) Circuit diagram based on the flexible thermoelectric generator, including power management with booster and capacitor for storing energy, and sensory system. Reproduced with permission.^[^ ^131^ ^]^ Copyright 2020, Elsevier. (f) Sweat information was obtained through the NFC function of the TENG device. Reproduced with permission.^[^ ^38^ ^]^ Copyright 2023, Wiley‐VCH GmbH. (g) Wireless NFC sensor and smartphone communication in real‐time. Reproduced with permission.^[^ ^132^ ^]^ Copyright 2021, Elsevier.

As the simplest and most effective conditioning circuit, a full‐wave rectifier is commonly used to convert the bi‐directional voltage input of TENGs into a direct pulsating output voltage. Song et al. proposed a battery‐free, fully self‐powered wearable system consisting of an efficient wearable freestanding mode TENG (FTENG), low‐power wireless sensor circuitry, and a microfluidic sweat sensor patch.^[^ ^36^ ^]^ As shown in **Figure** **11**a, to optimize power management, a commercial energy harvesting power management integrated circuit was used to manage the power generated by the FTENG with minimal power waste. With the aid of a bridge rectifier that converts the high‐voltage AC signal generated by the FTENG into a DC signal, the power management integrated circuit (PMIC) stores the power generated by the FTENG in 2 capacitors (220 and 22 µF) connected in parallel. Three SET_V_OUT_ resistors set programmable thresholds and hysteresis voltages to release the stored power only when absolutely necessary via the built‐in switching control logic. When powered by the storage capacitors, the load/output voltage is regulated by a voltage regulator to 2.2 V, providing a stable voltage for accurate measurement circuitry. In Figure 11b, the PENG‐based self‐powered circuit design integrates various electronic units for signal collection, processing, and transmission, including power management, voltage regulators, instrumentation amplifiers, microcontroller units, analog‐to‐digital converter ports, and Bluetooth modules.^[^ ^23^ ^]^ The power management unit controls electric flux into the device, i.e., when the voltage of its built‐in capacitor reaches a threshold, it shuts down the circuit and releases the current. The power management based on the PENG mode was summarized as PVDF as the energy generation module. After rectification, the electricity was stored in the capacitor with 5  ×  1000 µF capacity. When its voltage reaches a certain threshold, the released current powers the Bluetooth module and the instrument amplifier, and then transmits the processed signal to the user interface.

BFCs can obtain a DC output, but their relatively low voltage output cannot directly drive electronic devices such as LED displays. Therefore, additional energy management modules are required for current conversion. Figure 11c demonstrated a fully perspiration‐powered integrated electronic skin device consisting of a nanoengineered BFCs array, boost converter, biosensor array, instrumentation amplifier, and programmable system‐on‐chip module (integrated with the BLE module, microcontroller, and temperature sensor).^[^ ^35^ ^]^ The DC‐DC boost converter amplifies the signal potentials with low power loss (−20%). The output signal (3.3 V) continuously charges a capacitor (660 µF) that temporarily stores energy and powers the biosensors and other electronic components. PVs convert light energy into a DC voltage output, which is then charged to a supercapacitor or battery, providing a continuous and stable power supply that eliminates interference from ambient light. In order to build a self‐charging and self‐powering energy storage collection hybrid device, an integrated device of supercapacitor and flexible solar cell (0.3 W, 2 V) was proposed, as shown in Figure 11d.^[^ ^130^ ^]^ A DC‐DC boost converter was connected to the power supply to improve the driving capability of the system. A Schottky diode with a low forward voltage (1N5817, 20 V, 1 A) was connected between the supercapacitor and the solar cell to prevent the current from flowing backward from the interdigitated solid‐state supercapacitors (ISSCs) to the solar cell when the light intensity was low. Figure 11e depicts the power management of a wearable smart bracelet powered by a flexible thermoelectric generator (f‐TEG).^[^ ^131^ ^]^ The output voltage of the f‐TEG was boosted to 2.8–3.3 V by the booster for the power supply of electronics and sensors. A low‐power microcontroller was realized by using low power consumption single‐chip microcomputer, dynamically adjusting the power supply of sensors and liquid crystal display (LCD) to optimize resource allocation. In standby mode, the f‐TEG provides only minimal power to the accelerometer (5.4 µW) and LCD (9.5 µW), while the remaining energy is stored in the capacitor. In wake mode, the power from the f‐TEG is supplied to the entire sensory system (≈2 mW). Therefore, by adjusting the switching frequency between the standby mode and the wake mode, the use of the collected energy could be optimized.

### 4.3 Wireless Transmission and Data Analysis

The integration of wireless data transmission devices into self‐powered sweat biosensors, and the development of smartphone applications for data visualization and analysis. This allows users to easily view their physiological information, which contributes to the practicality and intelligence of wearable sensing devices. To repeat battery charging requirements, battery‐free data transmission/power supply methods such as near‐field communication (NFC) and Bluetooth Low Energy (BLE) are urgently needed.

The NFC was used to transmit measurement data wirelessly to smartphones, effectively alleviating the problems of large size, high energy consumption, and environmental pollution of commercial lithium‐ion batteries. Figure 11f illustrates the flowchart of a self‐powered monitoring system designed for sedentary elderly people and based on wireless microelectronics for data communication, processing, and collection based on NFC.^[^ ^38^ ^]^ For example, tapping the TENG device on their arm would collect the energy generated by the local movement, and the data is transmitted wirelessly via NFC. The sensor patch converts the collected data into digital signals and stores them in memory, which is transmitted to the mobile terminal via the NFC chip. The smartphone can display and store the received data for further analysis. To detect K^+^ concentration in human sweat in the field, a flexible electrochemical sensor was integrated and assembled with a low‐power RF energy harvesting battery‐free NFC wireless patch system. As shown in Figure 11g,^[^ ^132^ ^]^ the SD14 module with integrated NFC chip is a multi‐channel sigma‐delta analog‐to‐digital converter (ADC), [K^+^] and the output of the sensor was read through a 14‐bit ADC converter. A smartphone application was developed based on the Android studio software program to display the K^+^ concentration by reading and calibrating the output voltage of the ADC.

In terms of wireless data transmission, NFC has the characteristics of high security and fast transmission speed, but it requires a very close reading distance. The use of BLE transmission alleviates precisely these limitations and provides a more realistic communication solution for skin/wear applications.^[^ ^133^ ^]^ As shown in Figure 11a, efficient power management is matched with low‐power measurements through a low‐power instrumentation amplifier with a shutdown mode and low‐power data transmission via connectionless BLE advertisements.^[^ ^36^ ^]^ When the storage capacitor was charged to 3.5 V, the BLE programmed system on a chip (PSoC) module initiated an operation cycle of ≈510 ms, during which the 2 instrument amplifiers were awakened via general‐purpose input/output, 32 potential measurements were taken and averaged using a 12‐bit ADC, and then the amplifiers were turned off to reduce power consumption. After that, the PSoC goes into deep sleep mode for 500 ms, consuming ≈2 µA of current. Finally, the measurement results were sent to a nearby device via BLE. Throughout the process, the circuit consumed an average of 330 µA of current in 510 ms. Similarly, BLE advertising packets are widely favored in BFC‐type self‐powered sensors due to their small size of the data packets and low power consumption. In Figure 11c, the BLE module operates in bursts of activity, periodically waking up from deep sleep mode to acquire measurements with the embedded successive‐approximation analog‐to‐digital converter then wirelessly broadcasting the data to the user interfaces.^[^ ^35^ ^]^

### 4.4 Low Power Design

Self‐powered sweat sensor solves the problem of traditional wearable devices relying on an external power supply, have the characteristics of high efficiency, are non‐invasive and convenient, and provide strong technical support for the development of wearable health monitoring devices. **Table** **4** demonstrates the performance of different energy harvesters for powering wearable sweat sensing.

**Table 4:** Application of different energy harvesters in wearable sweat sensors.

Energy harvester	Energy Source	Output performance	Analyte	Stable operation time	Refs.
TENGs	Human movement	*P*_max _= 84 µW	Glucose	/	[89]
	Human movement	*P.D* = 2.5 W m^−2^	Lactate	/	[134]
	Human movement	*/*	Lactate	/	[17]
	Human movement	*P.D* = 0.774 mW cm^−2^	Na^+^	/	[135]
			K^+^		
	Human movement	*P.D* = 416 mW m^−2^	Na^+^ pH	60 min	[36]
				
	Human movement	**/**	Na^+^	/	[20]
			K^+^		
			Ca^2+^		
	Human movement	*P.D* = 0.44 W m^−2^	Na^+^	>5 h	[38]
			K^+^		
			pH		
	Human movement	*P.D* = 24.08 W m^−2^	Sweat level	/	[136]
PENGs	Human movement	**/**	Lactate	>30 min	[102]
			Glucose		
			UA		
			Urea		
	Human movement	*V_oc_* = 56 V	Cl^−^	> 50 min	[137]
			pH		
	Human movement	*P.D* = 2.7 mW m^−2^	K^+^	>35 min	[115]
			Na^+^		
	Joint movement	*P.D* = 140 mW m^−^^2^	Na^+^	>150 min	[23]
			K^+^		
			pH		
TEGs	Body heat	*P.D* = 53.9 mW m^−^^2^	Glucose	/	[24]
	Body heat	*P = *330 µW	pH	> 4.5 min	[138]
	Body heat	*P.D*_max_ = 42.3 mW m^−2^	Na^+^	/	[99]
			K^+^		
			Ca^2+^		
BFCs	Glucose	*P.D = *2.16 µW cm^−2^	Glucose	/	[26]
	Lactate	*P.D = 39.5 µW cm^−2^*	Lactate	/	
	Glucose	/	Glucose	15 h	[76]
	Glucose	*P.D* = 2.512 µW cm^−2^	Glucose	/	[79]
	Glucose	*P.D* = 160 µW cm^−2^	Glucose		[37]
	Lactate	*P.D* = 250 µW cm^2^	Lactate	50 min	
	Lactate	*P.D* = 13 µW cm^−2^	Lactate	/	[27]
	Lactate	*V_oc_* = 0.5 V	Lactate	24 min	[139]
	Lactate	*P.D* = 3.5 mW cm^−2^	NH_4_^+^	60 h	[35]
			Urea		
			Glucose		
			pH		
PVs	Solar, Zn‐MnO_2_ battery	*C *= 301 mA h g^−1^	Glucose	8 h	[28]
	Solar, Zn‐MnO_2_ battery	*C *= 190 mA h g^−1^	pH	/	[30]
TENGs & PENGs	Biomechanical	*P.D *= 11 µW cm^−2^	Sweat level	/	[116]
		*P.D *= 128 µW cm^−2^			
TENG & EMG	Biomechanical	*P_max_ *= 81 µW	Na^+^	/	[32]
		*P_max_ *= 43.5 mW	K^+^		

C: capacity; EMG: electromagnetic generator; P.D: power density; V_oc_: open‐circuit voltage.

As shown in Table 4, the self‐powered sweat sensor provides electrical support to the sweat‐sensing system by collecting and converting energy (e.g., human movement, body heat, chemicals in body fluids, solar energy). The energy consumption of sweat sensors mainly includes the detection function, signal processing, data transmission, and display output. Specific energy consumption values are not usually provided directly in articles, but it can be inferred that the design of self‐powered sweat sensors focuses on minimizing energy consumption. For example, in the design of the TENG, we can store and release the electrical energy generated by the TENG through an optimized power management integrated circuit.^[^ ^36^ ^]^ Low‐power instrument amplifiers and BLE modules are used for data transmission to reduce energy consumption. Optimize system energy consumption, when the storage capacitor is fully charged, the system performs a single run, disconnecting from the load before the capacitor is charged back to 3.5 V.

When using the energy collected by the BFCs, the energy is first extracted from the battery through a DC‐DC boost converter and stored in the capacitor.^[^ ^35^ ^]^ The system uses a low‐power analog‐to‐digital converter and Bluetooth low‐power module to run. With a periodic wake‐up strategy, the sensors and communication modules are activated only when needed, and the rest of the time they are in deep sleep mode to reduce energy consumption. In the hybrid power supply mode (such as TENG & EMG), energy management, signal processing, and wireless communication are realized through integrated power management modules, sensing analog circuits, and BLE modules.^[^ ^32^ ^]^ The system only releases energy for operation when the capacitor voltage reaches 3.3 V and stops the power supply when the voltage drops to 2.2 V to reduce energy waste. Therefore, through low‐power design (such as Bluetooth modules, signal processing modules, and system operation), energy conversion and storage, and energy conversion efficiency optimization, the sensor can operate for a long time under limited energy supply, providing reliable support for sweat monitoring.

## 5 Self‐Powered Sweat Sensor Applications

The biomolecules in human sweat contain a wealth of physiological information, and non‐invasive monitoring of the human body can be achieved by analyzing sweat composition. Emerging wearable energy harvesters have facilitated the development of biosensors, bringing a sustainable energy supply for personalized healthcare. The electrical signals are obtained from the energy collector as an indicator of physiological events or provide a power supply for biosensors, and support the operation of wireless data transmission and data analysis operations. This greatly simplifies the resulting sensor network, making wearable sweat‐sensing devices more feasible for a wider range of applications.

In terms of wearable sweat‐sensing energy supply, each energy harvesting technology has its own features and shortcomings. For example, TENG is flexible and stretchable, suitable for integration in wearable devices; The high output voltage makes it suitable for powering low‐power sensors. However, the energy conversion efficiency and stability of the device are affected by environmental factors (such as humidity), and the stability and durability in contact with sweat need to be further studied. PENGs exhibit high energy density and ideal electromechanical stability. The device has a fast response speed and is suitable for dynamic energy collection. At the same time, PENGs require the continuous action of external mechanical forces when powered, limiting the continuity of their application. There are also problems of low piezoelectric output power and structural instability of the device under long‐term mechanical stress. The TEGs convert the temperature difference of the human body into electricity without an additional power supply and are not affected by the external environment. Unfortunately, its thermoelectric conversion efficiency is low, requiring a large temperature difference to produce usable electricity. And the integration and compatibility of TEGs with wearable devices still need to be improved. In contrast, BFCs can realize the dual function of sweat detection and power supply, directly using biological molecules (such as glucose or lactic acid in sweat) as fuel, with high energy density and suitable for long‐term continuous power supply. However, its stability and durability are restricted by the complexity of the sweat environment, and long‐term exposure to sweat will affect its performance and service life. Solar energy is a renewable energy source, so PVs have the characteristics of environmentally friendly and renewable. The resulting power supply performance of PVs is limited by light conditions and low energy conversion efficiency. Composite energy harvesters, also known as hybrid energy harvesting systems, provide a solution to improve energy efficiency and system reliability in wearable sweat energy supply applications. But it also brings challenges in terms of system integration, energy management, and wearability.

Therefore, when selecting an energy harvester suitable for a wearable sweat‐sensing energy supply, factors such as energy density, stability, environmental adaptability, and compatibility with human sweat need to be considered. This section describes the application of self‐powered technology based on different energy harvesters in sweat biosensors, covering glucose sensors, lactate sensors, electrolyte sensors, pH sensors, and multifunctional sweat sensors.

### 5.1 Glucose Sensors

**Figure 12:**
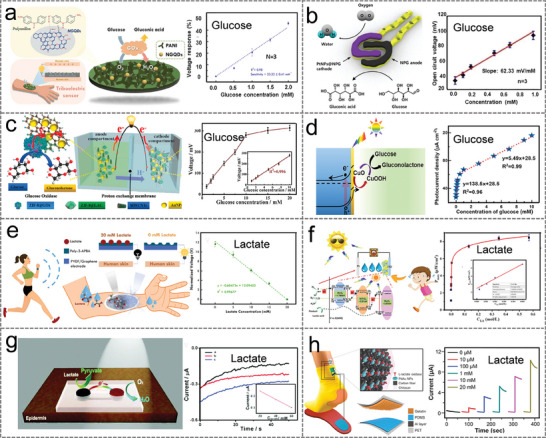
a) Self‐powered triboelectric sensor based on N‐doped graphene quantum dots for glucose detection. Reproduced with permission.^[^ ^89^ ^]^ Copyright 2023, Elsevier. b) Sensor patches based on self‐powered nonenzyme fuel cells for sweat glucose detection. Reproduced with permission.^[^ ^79^ ^]^ Copyright 2022, Wiley‐VCH GmbH. c) Self‐powered glucose biosensor based on enzyme biofuel cells. Reproduced with permission.^[^ ^76^ ^]^ Copyright 2021, Elsevier. d) Enzyme‐free sweat glucose photoelectrochemical sensing. Reproduced with permission.^[^ ^140^ ^]^ Copyright 2023, Elsevier. e) Self‐powered molecularly imprinted polymers‐based triboelectric sensor for noninvasive lactate monitoring in human sweat. Reproduced with permission.^[^ ^17^ ^]^ Copyright 2022, Elsevier. f) Photo‐fuel cell for lactate detection. Reproduced with permission.^[^ ^84^ ^]^ Copyright 2024, Elsevier. g) Wearable sensing energy device based on photoelectric biofuel cells for lactate analysis. Reproduced with permission.^[^ ^141^ ^]^ Copyright 2017, The Royal Society of Chemistry. h) Wearable self‐powered lactate sensor. Reproduced with permission.^[^ ^134^ ^]^ Copyright 2017, Elsevier.

Diabetes is one of the most common diseases in the world, and glucose is an important biomarker for diagnosing diabetes. Studies have shown that there was a relationship between glucose concentration in sweat and blood sugar levels, which provides a scientific basis for non‐invasive, wearable glucose detection.^[^ ^81^, ^82^ ^]^ **Figure** **12**a shows a novel non‐invasive wearable self‐powered triboelectric sensor for the non‐invasive detection of glucose in human sweat.^[^ ^89^ ^]^ Based on an N‐doped graphene quantum dot modified polyaniline nanocomposite (NGQDs/PANI) as a friction electrode layer, the device offered higher sensitivity for glucose detection (23.52 mM^−1^) than that of pristine PANI/GOx (16.44 mM^−1^). In practical applications, the sensor was connected to the LED circuit, and when the glucose concentration in sweat was too high (> 2 mM), the LED lights up to realize a self‐powered blood glucose monitoring alarm. Biofuel cells serve the dual function of providing electrical power to the device and detecting biomarkers. Figure 12b illustrates a stretchable, self‐powered sweat glucose sensor based on a non‐enzymatic fuel cell.^[^ ^79^ ^]^ Nanoporous gold was used as the anode to oxidize glucose to gluconic acid, and nanoporous gold‐coated platinum nanoparticles were used as the cathode to reduce O_2_ to H_2_O. The device detected glucose in the range of 0–1 × 10^−3^ M, with a sensitivity of 62.33 mV mM^−1^ and a detection limit as low as 9 × 10^−6^ M. By placing a glucose sensor patch on the back of a subject's hand or forehead and connecting it to a portable potentiometer, glucose levels in sweat can be detected in real‐time.

In addition, cellulose acetate/ZIF‐8@glucose oxidase/multiwalled carbon nanotubes/Au and cellulose acetate/ZIF‐8@laccase/multiwalled carbon nanotubes/Au nanostructured electrodes serve as anode and cathode to establish a self‐powered biosensor platform to monitor sweat glucose (Figure 12c).^[^ ^76^ ^]^The glucose biosensor demonstrated good linearity with glucose concentration in the range of 1–10 mM, with a detection limit of 5.347 µM (S/N = 3), and showed significant enhancement of long‐term stability with up to 15 h of continuous operation. As a new type of self‐driven sensor, photoelectrochemical (PEC) sensors operate by converting solar energy without the electrical bias, which is expected to enable simplified and portable detection of sweat glucose concentration. Figure 12d presented self‐driven and enzyme‐free sweat glucose PEC sensors based on TiO_2_ hierarchical nanotubes modified with CuO nanoparticles (CuO@TiO_2_ HNT).^[^ ^140^ ^]^ The sensitivity of the as‐prepared PEC sensors was138.9 µA mM^−1^ cm^−2^ in the low concentration range (<200 µM), with a detection limit of 0.7 µM (S/N = 3) at relatively zero bias.

### 5.2 Lactate Sensors

Lactate is a metabolite normally found in sweat, has been proved to be an indicator of performance in athletes during high‐intensity and endurance‐type activities. An imbalance in lactate production may manifest itself in respiratory failure, sepsis, hypoxia, lactic acidosis, and other abnormalities.^[^ ^142^ ^]^ Currently, biosensors for lactate detection in sweat face challenges such as expensive material usage, non‐removable power supply, and complex circuit connections, making it difficult to realize compact and sustainable sensing systems. To address these limitations, a self‐powered molecularly imprinted polymer‐based triboelectric sensor (MIP‐TES) was proposed to provide a versatile and noninvasive method for the specific and simultaneous detection of lactate (Figure 12e).^[^ ^17^ ^]^ The MIP lactate sensor showed excellent selectivity in lactate detection with a sensitivity of 5.18 µA cm^−2^ mM^−1^. Meanwhile, the MIP‐modified lactate sensor was further introduced into the friction nanogenerator system to convert mechanical energy into electrical energy output. By integrating MIP‐TES with LED, the LED can light up without any external power supply, successfully verifying the feasibility of the sensor on the surface of human skin. Zhou et al. established a self‐powered sensing platform with enzyme‐free photo‐fuel cells for lactate sensing using photoelectric conversion technology (Figure 12f).^[^ ^84^ ^]^ The NiTiO_3_/Bi_2_O_3_/MoS_2_ double Z‐type heterojunction system acts as the anode of the photo‐fuel cell to accelerate the electron transfer and improve the light absorption and photocatalytic efficiency, thereby improving the detection performance of lactate. The results showed that the prepared lactate sensor had a wide detection range (30 µM–5 mM), low detection limit (6 µM), high selectivity (8 µA µM^−1^ cm^−2^) and long‐term stability.

Photovoltaic biofuel cells (PBFCs), which convert light energy and biomass into electricity, and can simultaneously monitor ambient light and lactate, have been proven to be an essential device for monitoring metabolic production to reflect body function.^[^ ^143^, ^144^ ^]^ A self‐powered wearable sensing energy device (WSED) was designed based on the assembly of a PBFC with a lactate oxidase‐modified bioanode and photocathode covered by organic semiconductor polythiophene (Figure 12g).^[^ ^141^ ^]^ The WSED demonstrated excellent detection performance, monitoring lactate concentrations from 20 to 60 mM and responding to varying illuminances from 4150 to 37 600 lux with a detection limit of 1.04 lux. Improving the sensitivity of self‐powered sensing systems by introducing nanoparticles has become a common approach. Figure 12h illustrates the successful use of a TENGs‐based self‐powered electrochemical system for metal nanoparticle synthesis and lactate detection.^[^ ^134^ ^]^ With the operation of the TENGs for less than 1 min, the amount of electricity generated was enough to detect the concentration of lactate accumulated in human sweat in real‐time.

### 5.3 Electrolyte Sensors

**Figure 13:**
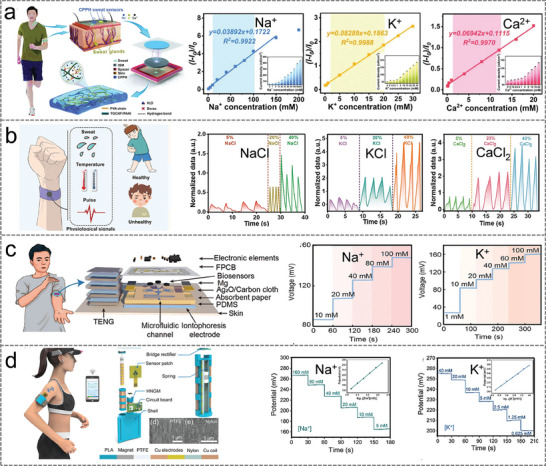
a) A fully flexible self‐powered sweat sensor for the detection of Na^+^, K^+,^ and Ca^2+^ in sweat. Reproduced with permission.^[^ ^20^ ^]^ Copyright 2022, Wiley‐VCH GmbH. b) Multimodal electronic skin based on flexible thermoelectric PLG hydrogel. Reproduced with permission.^[^ ^99^ ^]^ Copyright 2024, American Chemical Society. c) Triboelectric nanogenerator enabled sweat extraction and power activation for sweat monitoring. Reproduced with permission.^[^ ^38^ ^]^ Copyright 2022, Wiley‐VCH GmbH. d) A self‐powered wearable sensor for continuous wireless sweat monitoring. Reproduced with permission.^[^ ^32^ ^]^.Copyright 2022, Wiley‐VCH GmbH.

Electrolyte levels in biological fluids are key biomarkers for assessing health status and provide early warning information for a variety of diseases. For example, the concentration of ions including Na^+^, K^+^, and Cl^−^ are markers of dehydration during exercise activities. **Figure** **13**a demonstrates a fully flexible self‐powered sweat biosensor fabricated from a cellulose‐based conductive hydrogel.^[^ ^20^ ^]^ Considering the dynamic nature of sweat and its complexity, highly selective ion‐selective membranes (ISM) are used to separate specific sweat ions. The self‐powered sensor was assembled by the flexible electrode and ISM, and the content of Na^+^, K^+^, and Ca^2+^ in sweat was quantitatively determined via triboelectric effect, with a sensitivity of 0.039, 0.082, and 0.069 mmol^−1^, respectively. In practical applications, self‐powered sweat sensors provide continuous monitoring and data transmission of Na^+^, K^+^, and Ca^2+^ concentrations in sweat during exercise. Then, the wireless transmission technology was used to transmit the information output from the sensor to the mobile phone application. In addition, a passive multimodal e‐skin based on thermoelectric hydrogel was proposed to monitor multiple physiological signals using synergistically coupled sensing and transduction (Figure 13b).^[^ ^99^ ^]^ Due to the thermal diffusion of ions on the temperature gradient, the current response of the e‐skin to Na^+^, K^+,^ and Ca^2+^ in sweat tends to increase with increasing ion concentration. The gel‐based electronic skin could be worn directly on the wrist without further decoupling to satisfy the need for real‐time passive monitoring of body temperature, pulse, and sweat.

Despite the ease of access to sweat, its use was still limited due to the inherent inaccessibility of sedentary humans. Xu's research group designed a passive sweating strategy and self‐powered monitoring system (Figure 13c).^[^ ^38^ ^]^ The system utilized TENG to extract sweat and a sweat‐activated battery as an integrated power source. By clicking on the TENG, a sedentary person passively extracts sweat based on the ion electrophoresis process, allowing sensors to detect biological information in the sweat. The ion sensor consisting of poly (3,4‐ethylenedioxythiophene: poly(4‐styrenesulfonic acid sodium salt) (PEDOT: PSS) exhibited a linear correlation between the output voltage values of the sensor and the ion concentration in the ranges of 10–100 mM (Na^+^) and 1–100 mM (K^+^), with good anti‐interference ability and selectivity. In practical applications, by placing the TENG device on the subject's arm to achieve sweat collection and battery activation, the sensor was powered by the battery, and the data was transmitted wirelessly via NFC to facilitate and effectively achieve continuous health monitoring. In Figure 13d, a wireless self‐powered wearable sweat analysis system was assembled by supplying electrical energy through a hybrid nanogenerator.^[^ ^32^ ^]^ An ion‐selective electrode was constructed using a PEDOT: PSS membrane, the Na^+^ and K^+^ sensors show near‐Nernstian sensitivities of 67.43 and 30.42 mV decade^−1^ of ion concentration, respectively. As a proof of concept, volunteers were fitted with the device during running and jumping rope activities, and the system was able to respond within minutes and continuously monitor the concentration of electrolytes in sweat, such as Na^+^ and K^+^, and the measurements were consistent with the normal physiological range.

### 5.4 pH Sensors

**Figure 14:**
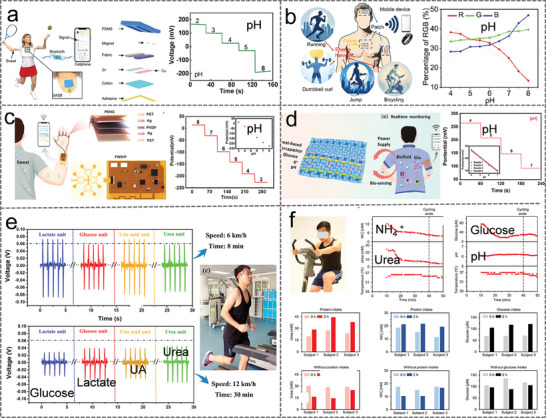
a) Sweat‐activated battery for wireless sweat components monitoring. Reproduced with permission.^[^ ^148^ ^]^ Copyright 2022, Wiley‐VCH GmbH. b) Self‐powered wireless sweat analysis patch based on pH determination by colorimetry. Reproduced with permission.^[^ ^137^ ^]^ Copyright 2024, Elsevier. c) Self‐powered sweat sensor based on piezoelectric nanogenerators and current response to pH. Reproduced with permission.^[^ ^23^ ^]^ Copyright 2022, Elsevier. d) Multifunctional fiber for synchronous biosensing and power supply in a sweat environment. Reproduced with permission.^[^ ^103^ ^]^ Copyright 2023, Wiley‐VCH GmbH. e) A practical application of self‐powered wearable non‐invasive electronic skin for sweat analysis. Reproduced with permission.^[^ ^102^ ^]^ Copyright 2017, American Chemical Society. f) Perspiration‐powered soft electronic skin for multiplexed wireless sensing. Reproduced with permission.^[^ ^35^ ^]^ Copyright 2020, The American Association for the Advancement of Science.

The pH value in sweat reflects an individual's hydration status, and monitoring sweat pH during physical activity is essential to assess body hydration, electrolyte balance, and muscle fatigue.^[^ ^145^, ^146^, ^147^ ^]^ Batteries are the most used power source in wearable electronic devices. However, batteries consist of hazardous substances and are bulky, which limits their incorporation into state‐of‐the‐art skin‐integrated electronics. Sweat‐activated batteries (SABs) offer a new powering strategy for skin‐like electronic devices. A soft, biocompatible, SABs were developed that can be directly integrated on skin with a high capacity of 42.5 mA h and power density of 7.46 mW cm^−2^ (**Figure** **14**a).^[^ ^148^ ^]^ The pH sensor based on polyaniline (PANI) has a sensitivity of 58.27 mV pH^−1^ and only responds to H^+^ concentration, which has good selectivity and anti‐interference ability. Traditional sweat detection uses benchtop instruments to collect, transfer and analyze samples, which are susceptible to contamination and sample loss and lack real‐time monitoring during physical activity. During strenuous cycling, the volunteers wore sensors that effectively monitored the dynamics of Na^+^, pH and glucose in their sweat. The measured biomarker information is wirelessly transmitted to a paired mobile phone for real‐time display. Cao and his colleagues reported a self‐powered wireless sweat‐analysis patch for personalized health monitoring (Figure 14b).^[^ ^137^ ^]^ The patch uses a piezoelectric unit to convert the mechanical energy generated by human movement into electrical energy, and a colorimetric method to analyze the content of target analytes (e.g., chloride ions and pH) in sweat. The colorimetric sensor uses a mixture of indicators (bromothymol blue, bromocresol purple, and thymol blue) to detect pH changes in sweat. In a practical application, the sensor was worn by the test subject while performing stationary cycling exercises and can monitor the chloride and pH levels in sweat in real‐time.

In Figure 14c, inspired by human joints as a biomechanical energy source, a battery‐free sweat sensing system integrating a self‐sustainable energy supply and a wireless communication interface was designed.^[^ ^23^ ^]^ Piezoelectric nanogenerators efficiently convert biomechanical energy from freely moving joints into electrical energy. For pH analysis, the pH ion‐selective electrode (ISE) was modified by mounting a HAuCl_4_ layer and a polyaniline layer, respectively, on the Au electrode to achieve H^+^ sensitivity. The pH sensor displayed near‐Nernstian sensitivities of 55.44 mV decade^−1^ concentration at room temperature. Smart fibers are ideal candidates for the fabrication of non‐invasive and conformal bioelectronic devices. As depicted in Figure 14d, a multifunctional textile patch based on reduced graphene oxide (rGO)/tetraphenylamine (TANi) fiber was designed for simultaneous monitoring of biomarkers and energy supply.^[^ ^103^ ^]^ Based on the multiple electrochemical redox states and proton doping/dedoping properties of TANi, rGO/TANi hybrid fibers were combined as energy storage devices and biosensors in physiological environments. The intrinsic properties of proton doping and dedoping endowed TANi with high pH sensitivity. The device has a strong linear relationship between open‐circuit potential and pH ranging from 4 to 7, with a sensitivity of 56.8 ± 1.1 mV pH^−1^. The self‐powered sweat sensor enables wearable continuous monitoring through integrated smart textile patches, collecting and transmitting biomarker data such as pH, glucose, and K^+^ concentration in sweat to a smartphone. However, there are problems with sensing lag and real‐time monitoring, and it may not work effectively at low sweat rates.

### 5.5 Multifunctional Sweat Sensors

The emerging multifunctional flexible electronic skin is a new personalized medicine technique, which achieves real‐time monitoring of individual health status through the establishment of body‐electric interactions. Figure 14e demonstrated a self‐powered wearable noninvasive electronic skin for sweat analysis based on a matrix of enzyme/ZnO nanoarray piezoelectric biosensing units.^[^ ^102^ ^]^ The piezoelectric impulse of the piezo‐biosensing units as a power source and data biosensor and its working mechanism were attributed to the piezoelectric‐enzyme‐reaction coupling effect of enzyme/ZnO nanowires. As the test subjects ran at different speeds, the electronic skin applied constant and steady pressure through the fingers to detect piezoelectric signals through a low‐noise preamplifier. The results showed that the concentration of these 4 biomarkers in sweat increased with the the running time and intensity, especially the concentration of lactic acid. This was consistent with the fact that lactic acid was continuously produced during exercise. Yu et al. reported a battery‐free, fully sweat‐powered e‐skin that provides continuous multiplexed monitoring of critical metabolic biomarkers (e.g., NH_4_^+^, urea, glucose, and pH) via a lactate biofuel cell that derives energy from human sweat. As demonstrated in Figure 14f, in the biking exercise, the device was able to steadily harvest energy from human sweat and monitor key metabolites and skin temperature in real‐time, while transmitting data wirelessly via Bluetooth. During exercise, urea and NH_4_^+^ levels in sweat decrease rapidly and then stabilize; Glucose levels showed a similar downward trend, while pH remained stable. These results demonstrate the effectiveness of self‐powered sweat sensors in practical human applications, especially the potential for health and exercise monitoring. Marking the transition of self‐powered sweat sensors from the lab to life.

## 6 Conclusion and Outlook

Sweat biosensors are capable of non‐invasively detecting a variety of biomarkers, playing a crucial role in health monitoring and disease diagnostics. The introduction of self‐powered technology addresses the energy supply issue for wearable electronic devices. Furthermore, advances in material science, circuit integration, and multiplexed sensing modes are driving the rapid evolution of self‐powered sweat biosensors. At present, self‐powered sweat biosensors have been successfully promised for the detection of analytes such as electrolytes, glucose, and lactate. This has propelled self‐powered sweat sensors from the lab to life, providing innovative solutions for personal health monitoring.

### 6.1 Recent Advances in Wearable Sweat Sensors

**Figure 15:**
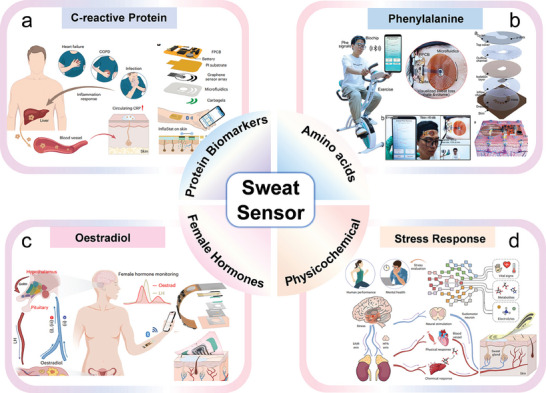
a) A wireless patch for the monitoring of C‐reactive protein in sweat. Reproduced with permission.^[^ ^149^ ^]^ Copyright 2023, Springer Nature. b) Wearable multimodal biochip for dynamic exercise sweat analysis evaluation. Reproduced with permission.^[^ ^52^ ^]^ Copyright 2024, Springer Nature. c) A wearable nanobiosensor for non‐invasive, non‐reagent analysis of female reproductive hormones. Reproduced with permission.^[^ ^150^ ^]^ Copyright 2023, Springer Nature. d) A physicochemical‐sensing electronic skin for stress response monitoring. Reproduced with permission.^[^ ^100^ ^]^ Copyright 2024, Springer Nature.

Recently, wearable sweat sensors have made breakthroughs in detecting low levels of disease markers (e.g., proteins, amino acids, and hormones) and stress, which provides a direction for the development of self‐powered sweat sensors in the future. The quantification of protein biomarkers in blood at picomolar‐level sensitivity requires complex manipulation, and there are significant interpersonal and individual variations in the composition of proteins sensing in sweat. As a result, real‐time sensitive analysis of proteins suffers from serious impediments. Tu's group reported a wearable wireless patch for real‐time electrochemical detection of the inflammatory biomarker C‐reactive protein (CRP) in sweat.^[^ ^149^ ^]^ As shown in **Figure** **15**a, it integrated iontophoretic sweat extraction, microfluidic channels, and a graphene‐based sensor array for quantifying CRP. Highly sensitive and efficient electrochemical detection of CRP at trace levels in the skin using gold nanoparticle‐decorated laser‐engraved graphene. The integrated graphene sensors measure pH, temperature, and ionic strength to calibrate CRP data in real‐time and on an individualized basis, reducing interpersonal sample error and providing a more comprehensive assessment of inflammatory status. Amino acids (AAs) are the building blocks of life, and AAs in sweat reflect their levels in the blood and are reliable biomarkers for assessing physiological status. Phenylalanine is an essential AA that is present in small amounts of sweat on the surface of the skin. In Figure 15b, an integrated wearable biochip for multimodal detection of phenylalanine and chloride concentrations and sweat loss levels (volume and rate) was presented.^[^ ^52^ ^]^ A unique phenylalanine sensing method based on phenylalanine‐imprinted enzymes mimicking molecularly imprinted polymers allows direct electrocatalytic oxidation of phenylalanine with high sensitivity and selectivity. A complex multipurpose microfluidic was designed to enable the acquisition of quantitatively multiple sweat metrics through scalable and low‐cost laser engraving techniques.

In situ monitoring of female hormones in sweat remains challenging due to extremely low concentrations (picomolar levels) and variations in sweat accessibility and sample matrix. Figure 15c demonstrated a skin‐interface wearable aptamer nanobiosensor based on target‐induced chain displacement for automated and noninvasive monitoring of estradiol by in situ sweat analysis.^[^ ^150^ ^]^ The flexible sensor contains a biorecognition interface modified by an estradiol‐selective deoxyribonucleic acid (DNA) aptamer facing gold nanoparticles‐MXene (AuNPs‐MXene) based detection working electrode that provides exceptional sensitivity with an ultra‐low detection limit of 0.14 pM. This fully integrated system controls precise microfluidic sweat sampling via capillary rupture valves, real‐time estradiol analysis, and calibration, simultaneous collection of multivariate information (i.e., temperature, pH, and ionic strength), as well as signal processing and wireless communication with the user interface. At present, electrochemical biosensors have become a promising tool for tracking human physiological dynamics through non‐invasive sweat analysis. However, integrating multiple sensors in a highly controllable and reproducible manner to achieve long‐term reliable biosensing still faces challenges. Approaches to quantify stress responses typically rely on subjective surveys and questionnaires. Xu's team reports an electronic skin for stress response assessment that non‐invasively monitors 3 vital signs (pulse waveform, galvanic skin response, and skin temperature) and 6 molecular biomarkers in human sweat (glucose, lactate, uric acid, sodium ions, potassium ions, and ammonium, which are closely related to the stress response) (Figure 15d).^[^ ^100^ ^]^ Using biochemical and physiological signals, real‐time multimodal data on stress responses were generated from 3 different stressors. With the help of a machine learning pipeline, the platform can distinguish between the 3 stressors with 98.0% accuracy and quantify psychological stress responses with a confidence level of 98.7%.

### 6.2 Prospects of Self‐Powered Sweat Sensors

**Figure 16:**
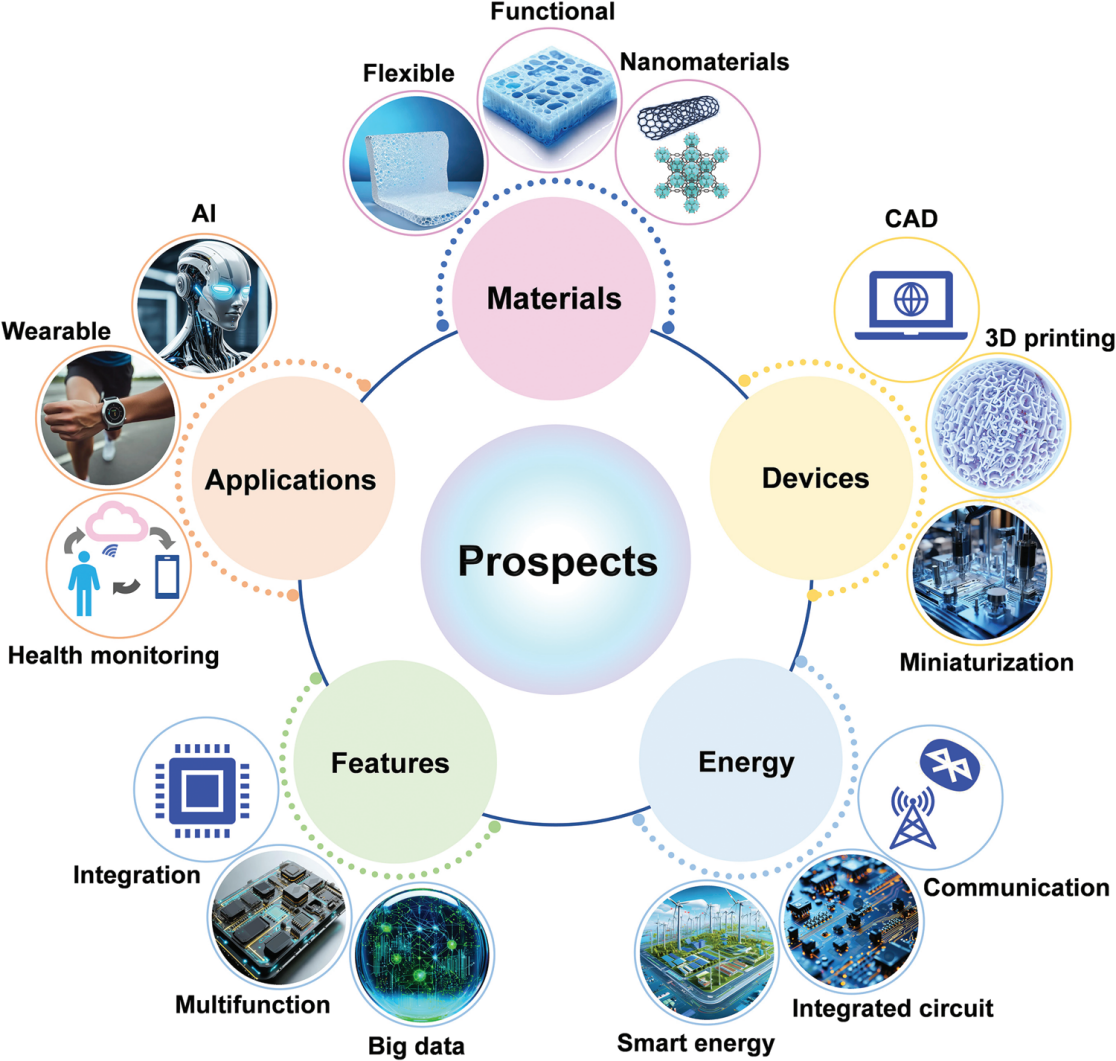
Prospects of self‐powered sweat sensors in materials, devices, energy, features, and applications.

Wearable sweat sensors have demonstrated the capacity for non‐invasive detection of physiological biosignals, encompassing a spectrum of biomarkers such as metabolites, electrolytes, and even trace levels of proteins, amino acids, and hormones. To achieve continuous and real‐time monitoring of human status, there is an urgent need for wearable biosensing systems with self‐powered capabilities. The integration of energy harvesters with wearable sweat biosensors is key to building self‐powered wearable sensor systems, heralding a new era in personalized healthcare. We systematically discussed the features and challenges of self‐powered sweat sensors in terms of sweat sensors, energy harvesters, energy management, and applications in the previous sections. Next, we provide an outlook on their future development direction from the perspective of health needs and practice transformation (**Figure** **16**).

#### 6.2.1 Materials

Novel materials play a critical role in improving sensor performance and energy harvesting efficiency. The selectivity, sensitivity, repeatability, stability, and mechanical reliability of the sensor are the essential indicators to measure its success in practical applications. Sweat is a complex environment, and the selectivity and sensitivity of sensors for low concentrations of biomarkers (e.g., proteins, hormones) at the pM level are particularly important. To improve the sensing performance and stability of the sensors, different forms of novel nanomaterials need to be developed. For example, molecularly imprinted polymers with specific receptors or recognition sites are designed to achieve highly selective recognition of specific biomarkers. High surface area nanowires, nanotubes, or nanoparticles are used to increase the surface‐active site of the sensor, thereby enhancing the detection sensitivity of low‐concentration biomarkers. The development of flexible materials, including carbon nanotubes, graphene, and conductive polymers for the manufacture of stretchable sensors and power supplies to improve the comfort and stability of wearable devices. In addition, the choice of materials directly affects the energy conversion efficiency of energy harvesters. In piezoelectric or friction electric sensors, the selection of materials with appropriate piezoelectric coefficients or friction electric properties (e.g., polyvinylidene fluoride, polytetrafluoroethylene, carbon material) can effectively increase the ratio of mechanical energy to electrical energy. Therefore, the development of new materials with good biocompatibility, mechanical stability, and excellent electrochemical properties is the key to advancing self‐powered sweat sensor technology and achieving widespread application.

#### 6.2.2 Device Miniaturization

The miniaturization of sensors is necessary to achieve the integration of their wearable and portable devices. Advanced design techniques like computer‐aided design (CAD) and finite element analysis (FEA) are utilized to create smaller sensor structures. Development of lightweight, flexible, and efficient energy harvesting and conversion materials, like high‐efficiency photovoltaic, piezoelectric, and friction electric materials, to build the foundation for the construction of miniaturized self‐powered devices. Miniaturization of devices by designing compact and efficient structures using nanotechnology and microelectromechanical systems technologies. Miniaturization and mass manufacturing of complex structures using advanced micro and nanomanufacturing techniques, as 3D printing, lithography, and flexible electronics manufacturing. For example, develop a micro‐sweat sensing array based on bio‐fuel cells that can be precisely embedded in a smartwatch or clothing to monitor electrolyte and metabolite concentrations in sweat in real‐time, providing athletes with real‐time physiological feedback. It is expected that in the future, nanoscale sensor units will be able to achieve accurate detection of multiple biomarkers.

#### 6.2.3 Energy Management

When designing wearable self‐powered sweat sensing devices, efficient energy harvesting, low‐power design, and smart power distribution are essential to achieve energy management. Advanced materials and technologies, such as friction electric or piezoelectric materials, are used to increase the efficiency of capturing energy from human sweat or movement. For instance, the development of new flexible battery technologies (lithium‐sulfur or lithium‐oxygen‐based batteries) to achieve higher energy densities. Hybrid energy systems using supercapacitors or batteries enable fast charging and discharging and high energy storage. Use low‐power communication technologies such that BLE, ZigBee for Narrowband Internet of Things, and Long Range to reduce energy consumption during communication. Sensors and circuits are designed to enter low‐power or sleep mode when no activity is detected. Designing low‐power sensors and circuits reduces unnecessary energy consumption and extends the life cycle of the device. Development of adaptive power management systems that dynamically adjust energy allocation based on the operating status and energy availability of the device to improve power utilization. As a result, self‐powered sweat sensors will be able to manage energy more efficiently and provide longer, more reliable health monitoring services, while reducing user maintenance costs and improving utility.

#### 6.2.4 Multifunction Integration

Combining multiple sensing technologies (e.g., electrochemistry, optics, electricity) into a single device not only detects electrolytes and metabolites in sweat but also monitors crucial vital signs such as heart rate, blood pressure, and body temperature. The device would be able to capture richer health information from a single sweat sample, providing a comprehensive snapshot of physiological status. Development of data fusion algorithms to integrate data from different sensors for a more comprehensive assessment of physiological status. Synergizing the integrated sensors with highly sophisticated circuitry and advanced signal processing algorithms ensures the accuracy of the monitoring data and the reliability of the multidimensional health metrics. This integrated design optimizes the functionality of the device and greatly improves user access to comprehensive health data.

#### 6.2.5 Intelligent Applications

Intelligent applications would revolutionize the user experience for sweat‐sensing devices. Integrated artificial intelligence (AI) algorithms, including machine learning and deep learning models, empower devices with powerful data processing and pattern recognition capabilities. The devices will be able to parse complex physiological data in real‐time to identify individual health trends and potential health risks, providing physical early warning to patients with chronic diseases (e.g., cardiovascular diseases, diabetes, and respiratory diseases). Designed intelligent wearable system with adaptive learning ability, dynamically adjusting monitoring parameters and data analysis model according to the user's physiological response and behavior pattern. For an example, the combination of self‐powered equipment and big data models can develop intelligent sports App software to provide personalized training suggestions for sports athletes and people's daily exercise. This intelligence would improve the accuracy of monitoring and enable the device to be seamlessly integrated into the user's daily life, becoming an intelligent partner in health management.

In summary, we have highlighted and reviewed recent developments in self‐powered sweat sensors, from wearable sweat sensors, and wearable energy harvesters to power management and applications of self‐powered sweat sensors. Wearable sweat biosensors were introduced, including detection methods, nanomaterials, and wearable devices. The working mechanism, composition structure, and application characteristics of different types of energy harvesters were highlighted. The features and shortcomings of sweat sensors based on different energy harvesters in energy management were analyzed in detail. The signal processing and data transmission of self‐powered sensing systems were studied for efficient energy utilization. Recently, self‐powered sweat sensors have made some achievements but still face significant challenges in real‐life and biomedical applications. In the foreseeable future, innovative materials, miniaturized devices, efficient energy management, integrated functionality, and intelligent applications will drive the development of self‐powered sweat sensors, offering promising insights for applications in personalized healthcare and smart medicine.

## Conflict of Interest

The authors declare no conflict of interest.
